# Genetic and morphological variation in the genus *Zygogonium* (Zygnematophyceae, Charophyta) from localities in Europe and North America and description of *Z. angustum*, sp. nov.

**DOI:** 10.1111/jpy.70012

**Published:** 2025-04-09

**Authors:** Rosalina Stancheva, Louise A. Lewis, John Hall, Tereza Šoljaková, Charlotte Permann, Andreas Holzinger

**Affiliations:** ^1^ Department of Environmental Science and Policy George Mason University Fairfax Virginia USA; ^2^ Department of Ecology and Environmental Biology University of Connecticut Storrs Connecticut USA; ^3^ Academy of Natural Sciences of Drexel University Philadelphia Pennsylvania USA; ^4^ Department of Botany, Faculty of Science Charles University Prague Czech Republic; ^5^ Department of Botany University of Innsbruck Innsbruck Austria

**Keywords:** *atp*B, Charophyta, conjugating green algae, Conjugatophyceae, phylogeny, plant terrestrialization, *psb*C, *rbc*L, streptophyte, ultrastructure

## Abstract

The globally distributed genus *Zygogonium* exhibits a narrow environmental range, with 19 morphologically described species. Its molecular characterization is poor, based on a single accession of the type species *Z*. *ericetorum* from Austria. We examined the genetic variability, morphology, and ultrastructure of field‐collected *Zygogonium* material from different sampling sites in Austria, Norway, Ireland, Scotland, and the United States. Phylogenetic analysis based on partial sequences of the *psb*C gene distinguished three well‐supported groups and one subgroup. *Atp*B gene sequences collected from a subset of samples also support this result, while *rbc*L gene data provided lower support. Group 1A contained the type species *Z. ericetorum* from Obergurgl/Austria and samples from Kühtai/Austria, Norway, and Scotland. The morphology was characterized by wide vegetative filaments (15–31 μm) and the occurrence of aplanospores with purple residue. Group 1B contained *Z.* cf. *ericetorum* from Ireland and Ellmau/Austria. Filaments were in a similar size range (12–30 μm) as in group 1A. This group had round unusual akinetes with green or purple content, had one or two chloroplasts, and was surrounded by a thick cell wall; no aplanospores were observed. Group 2 collected from Ireland had narrower filaments (8–12 μm), cells up to six times longer than wide, and contained elongated aplanospores. Therefore, we have described a new species *Z. angustum* sp. nov. Group 3 contained *Z.* cf. *ericetorum* from Norway and the United States, represented by vegetative filaments with an intermediate width (13–20 μm), but no other distinct morphological features. The morphological and genetic variability observed in *Zygogonium* is possibly related to habitat and ecology.

**Abbreviations**
*atp*B: ATP‐synthetase subunit B
BBM: Bold's Basal Medium
ML: maximum likelihood
MP: maximum parsimony
*psb*C: photosystem II CP43 gene
*rbc*L: RUBISCO large subunit
TEM: transmission electron microscope

## INTRODUCTION

The streptophyte green algal class Zygnematophyceae has recently gained much attention, as they are considered the sister lineage to land plants (de Vries et al., 2018; One Thousand Plant Transcriptomes Initiative, 2019; Wodniok et al., 2011) and the first genomes have been published for *Closterium* sp., *Mesotaenium endlicherianum*, *Penium margaritaceum*, *Spirogloea muscicola*, *Zygnema circumcarinatum*, and *Z. cylindricum* (Cheng et al., 2019; Feng et al., 2024; Jiao et al., 2020; Sekimoto et al., 2023). A novel, phylogenomically informed five‐ordered system has been suggested by Hess et al. (2022). They defined the orders Desmidiales, Spirogyrales, Zygnematales, Serritaeniales, and Spirogloeales (Hess et al., 2022). Previous phylogenetic studies on *Zygogonium* (Zygnematales) focused entirely on the type species *Zygogonium ericetorum* Kützing, 1843 (collected near Obergurgl, Austria, Stancheva et al., 2014), providing the only DNA sequences for this genus at the time (Stancheva et al., 2014). This study suggested a sister relationship to a not‐further‐characterized *Mesotaenium* sp. (JH0031; Hall et al., 2008; Stancheva et al., 2012, 2014). Busch and Hess (2022a) recently performed a more detailed investigation on underappreciated Zygnematophyceae and grouped *Zygogonium ericetorum* as sister to their lineage “Meso‐5” (out of 12 *Mesotaenium*‐like zygnematophyte lineages characterized by *rbc*L gene data). Meso‐5 contained two strains, which could not be assigned to an existing species with certainty, but which showed some similarities to the species *Mesotaenium truncatum* (West & West, 1904).

Many of these Zygnematophycean algae have a purple to brown pigmentation in common, resulting from the presence of different, mostly iron‐complexed, phenolic pigments. *Ancylonema*, a frequent member of snow microalgae communities, is known for its intense coloration (Peter et al., 2024). The responsible pigment has been shown to have a broad absorption in the visible light and UV range (Remias et al., 2012). Recently, its expression was experimentally induced by combining low‐nutrient conditions with ultraviolet B, providing further evidence for its UV screening properties (Busch, Slominski, et al., 2024). The newly described genus *Serritaenia* (Serritaeniales) also produces a conspicuous red pigmentation with sun‐shielding properties (Busch & Hess, 2022b). In *S. testaceovaginata*, UV stress exposure triggered the production of the pigment alongside the expression of genes salient to phenylpropanoid biosynthesis and downstream peroxidases, which suggests a polyphenolic origin of the purple pigment (Busch, Gerbracht, et al., 2024). Interestingly, the pigment in *S. testaceovaginata* does not accumulate in the vacuoles, as it does for other Zygnematophyceae, but is part of the extracellular mucilage (Busch & Hess, 2022b). The variety in the expression site of such pigments adds another layer of complexity, and we are only at the beginning of understanding their biosynthesis, triggering factors, and biological function.

In this regard, the genus *Zygogonium* has also attracted attention due to the conspicuous purple appearance of its mats observed in nature (Holzinger et al., 2010; Lynn & Brock, 1969). These observations have resulted in investigations of the chemical nature of the purple pigments, which have been characterized as ferric (gallate)_2_ complex, that is, part of a polysaccharide of variably linked and branched glucose monomers (Newsome et al., 2013; Newsome & van Breemen, 2012). This pigment also likely possessed UV‐shielding capacities (Aigner et al., 2013). However, in *Zygogonium*, in addition to the “purple morph,” a “green morph” lacking this pigmentation has been observed (Aigner et al., 2013). The green morph has been observed in nature, mostly in parts of the mat that are below the purple morph (Aigner et al., 2013), or as a result of incubation in standard culture conditions with low light and the exclusion of UV light (Herburger et al., 2016). Furthermore, the color of the filaments changes with the life‐cycle stages. Stancheva et al. (2014, 2016) showed that the purple compounds remained as residue outside the aplanospores and zygospores, which produced bright green young filaments upon germination. The photophysiological properties of *Z. ericetorum* have been described in samples collected from Yellowstone National Park, United States (Lynn & Brock, 1969); Tyrol, Austria (Aigner et al., 2013; Herburger et al., 2016; Holzinger et al., 2010); and Scotland (Herburger et al., 2016). When comparing the samples from Tyrol and Scotland, no significant differences in morphology or photophysiology were observed (Herburger et al., 2016). The genus *Zygogonium* has been described as having preferences for acidic habitats (Herburger et al., 2016; Hoppert et al., 2004; Lynn & Brock, 1969), which make the availability of toxic metals like iron and aluminum possible. Iron is complexed by phenolic compounds, whereas aluminum remains mostly in the cell walls (Herburger et al., 2016). Numerous physiological investigations have been performed with *Zygogonium* samples from different localities (e.g., United States, Yellowstone: Lynn & Brock, 1969; Austria, Tyrol: Aigner et al., 2013; Austria, Scotland: Herburger et al., 2016); however, it remains unknown if the analyzed samples belong to similar or distinct genotypes.

Very limited molecular data are available on this genus and are restricted to the well‐described type species *Zygogonium ericetorum* (Stancheva et al., 2014). In this species, a multi‐gene analysis has been performed, analyzing the *rbc*L, *psb*C, and *atp*B genes. Moreover, in this species, sexual reproduction features have been described (Stancheva et al., 2016). The description of the type species *Zygogonium ericetorum* made several taxonomic changes necessary, such that the species is defined as having (i) chloroplast morphology described as irregular, compressed plate‐shaped as well as (ii) residual cytoplasmic content left in sporangia after aplanospore formation. The authors transferred 18 former *Zygogonium* species to the genus *Zygnema* (Stancheva et al., 2014), and there are 19 taxonomically accepted *Zygogonium* species names (Guiry & Guiry, 2024).

The aim of the present study is to investigate the genetic and morphological variability of the genus *Zygogonium* from different biogeographical regions in Europe (Austrian Alps, Norway, Ireland, Scotland) and the United States. Our main objectives were to test if samples from different localities contain different genotypes and if a possible genetic variability is reflected in morphological features. The outcome of this study will be beneficial for a deeper understanding of the variability within the genus *Zygogonium* and future investigations on physiological parameters.

## MATERIALS AND METHODS

### Sampling localities

**FIGURE 1:**
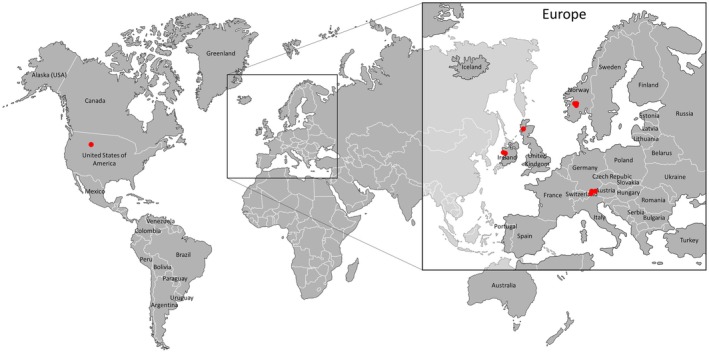
Map of *Zygogonium* sampling locations, including Austria (three sites, Obergurgl, Kühtai, Ellmau; Tyrol), Ireland (2 sites), Norway (three sites), and the United States (Tantalus Creek, Yellowstone National Park).

**FIGURE 2:**
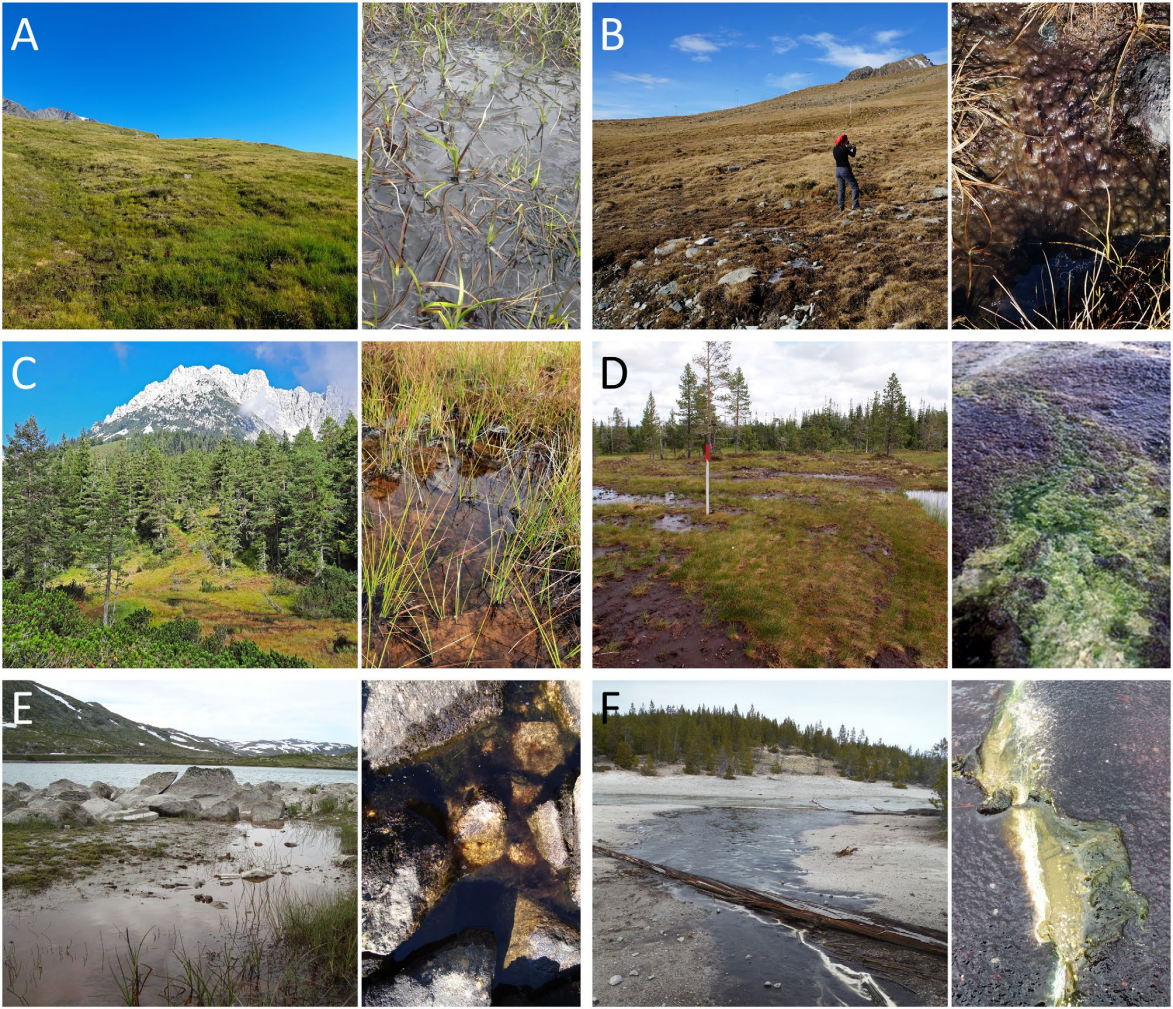
Habitats where *Zygogonium* samples were collected for the present study. In each panel, an overview (right) and a detailed view (left) is given. Obergurgl, Tyrol, Austria (a), Kühtai, Tyrol, Austria (b), Ellmau, Tyrol, Austria (c), Ireland 2–6 (d left) and Ireland 4–7 (d right), Norway (e), Tantalus Creek, Yellowstone National Park, United States (f).

Field samples with *Zygogonium* filaments were collected in different localities in Europe (Austria, Scotland, Ireland, Norway) and the United States (Yellowstone National Park), as illustrated in Figure 1 and summarized in Table 1. All sampled habitats in Europe were standing water, either shallow puddles of springs or peat bogs (Figure 2a–e), while the sample from the United States was collected from the shallow‐running Tantalus Creek (Figure 2f). *Zygogonium* field samples were collected at different times in the growing season (July 2013; August and November 2014; June, August, September, and November 2015) and had different degrees of moisture (Table 1). Water quality parameters (pH, conductivity, temperature) were measured with a field portable meter (WTW pH/Cond 340i). Fresh *Zygogonium* filaments were kept cold on ice and shipped to Rosalina Stancheva at California State University San Marcos (CSUSM) laboratory for morphological observations and selecting filaments for DNA extraction (see below). The sorted samples for DNA extraction were sent overnight to John Hall at the Academy of Natural Sciences, Philadelphia, United States.

**TABLE 1:** Locality information and reference DNA sample number for the studied *Zygogonium* samples. Legend: NA – not available. Note: Both samples from Ireland contain a mixture of *Z. ericetorum* and *Z. angustum* sp. nov. All other samples contain a single *Zygogonium* species.

Locality	Phylogeny Group	DNA Reference	*Zygogonium* Taxon	Habitat	Sampling Date	Coordinates	Altitude (m a.s.l.)	pH	Conductivity (μS/cm)	Temperature (°C)
Tyrol, Obergurgl, Austria	1A	JH1396, JH1397 Stancheva et al. (2014)	*ericetorum*	Standing shallow water among *Sphagnum* mosses	18 July 2013	46° 50′ 59.88″ N, 11° 0′ 54.18″ E	2350	4.6	NA	NA
Tyrol, Kühtai, Austria	1A	1516, 1517, 1518, 1519, 1520, 1521	*ericetorum*	Standing shallow water	03 November 2015	47° 13′ 20.4″ N, 11° 1′ 37.68″ E	2283	6.8	18	7.8
Norway 2	1A	1499, 1506	*ericetorum*	Standing shallow water	06 September 2015	60° 40′ 18.156′′ N, 8° 2′ 55.968″ E	951	7.6	13	10
Norway 3	1A	1509, 1510	*ericetorum*	Standing shallow water	06 September 2015	60° 32′ 52.224″ N, 7° 42′ 8.064″ E	1106	7.9	6	9.9
Lochaber, Scotland	1A	1477 Herburger et al. (2016)	*ericetorum*	Algal filaments covered by thin water layer	16 August 2014	56° 41′ 5.607″ N, 5° 5′ 25.778″ W	45	4.6	NA	16.9
Tyrol, Ellmau, Austria	1B	1479, 1480	*ericetorum*	Shallow puddle, very dry	07 June 2015	47° 31′ 58.752″ N, 12° 18′ 32.609″ E	1150	3.0	53.5	5.3
Ireland 4–7	1B	1482, 1491	*ericetorum*	Shallow puddle	03 August 2015	53° 28′ 28.801″ N, 9° 32′ 39.901″ W	53	6.6	46	15.5
**Ireland 2–6**	**2**	**1484, 1490, 1496, 1498**	***angustum*, sp. nov**	**Wet soil**	**01 August 2015**	**53° 27′ 16.999″ N, 9° 57′ 24.001″ W**	**22**	**7.2**	**148**	**18.3**
Norway 1	3	1507, 1508	*ericetorum*	Wet soil	05 September 2015	60° 41′ 16.400″ N, 8° 3′ 5.623″ E	NA	NA	NA	NA
Tantalus Creek, USA	3	1470, 1471, 1472, 1473, 1475	*ericetorum*	Running shallow creek	02 November 2014	44° 44′ 4.088″ N, 110° 43′ 2.605″ W	2299	3.0–3.5	NA	11.3

*Note*: Bold font indicates the newly described species.

### DNA extraction, PCR amplification, and sequencing

From morphologically characterized samples, several representative filaments were separated for DNA extractions. All field samples contained a single *Zygogonium* morphotype, except for both samples from Ireland, for which two distinct *Zygogonium* morphotypes were observed intermixed (narrow and wide filaments). The narrow *Zygogonium* filaments were selected for DNA extraction from field sample Ireland 2–6, while the wide *Zygogonium* filaments were collected for DNA extraction from sample Ireland 4–7. DNA was extracted with a Nucleon Phytopure DNA extraction kit (GE Healthcare, Pittsburgh, PA, United States) according to previously described methods (Hall et al., 2008; Stancheva et al., 2014). Portions of the chloroplast genes *psb*C, *atp*B, and *rbc*L were amplified using previously described primers (*psb*C_33F, *psb*C_547F, *psb*C_714R, *psb*C_1285R; *atp*B_175F, *atp*B‐672F, *atp*B_706R, *atp*B_1404R; *rbc*L_Z1, *rbc*L_526F, *rbc*L_635R, *rbc*L_1288R; Hall et al., 2008; Peréz et al., 2014; Stancheva et al., 2013). DNA sequencing was performed by Eurofins Genomics LLC (Louisville, KT, United States).

### Phylogenetic analysis

The phylogenetic analysis was based on partial or complete sequences of the *psb*C gene. For most investigated strains, *rbc*L gene sequences are available, and we collected *atp*B gene sequences from a smaller number of strains. Our data set was incomplete, as not all DNA extractions resulted in successful polymerase chain reaction (PCR) amplification or sequence data. For each specimen and gene, forward and reverse sequence reads were edited to remove low‐quality bases, then assembled into a consensus. These were compared to National Center for Biotechnology Information (NCBI) GenBank for nearest matches and aligned in Geneious. We performed Bayesian analyses using MrBayes 3.2.6 (Huelsenbeck & Ronquist, 2001). Trees were generated for each under the GTR model with gamma rate variation. Four heated chains of 1.1 million generations were specified, with a burn‐in of 100,000 generations, to yield summary trees with Bayesian posterior probability (BPP) values. We added a multiple likelihood (ML) analysis of the *psb*C gene data set, with 100 bootstrap replicates under the GTR + gamma model.

### *Zygogonium* isolation and culturing

Upon receiving the fresh samples at CSUSM, we picked single, healthy‐looking vegetative filaments with green chloroplasts and cultured them in Bold's Basal Medium (BBM, Nichols & Bold, 1965) on a 12:12 light:dark cycle at 12°C. We followed the same lab procedure as for previous successfully cultured *Zygnema* strains from streams in California (Stancheva et al., 2012). However, *Zygogonium* cells did not divide and were overgrown by other algae after 25 days in culture. We attribute the lack of success to light and temperature stress of filaments during several days of shipping overseas. We searched Algal Culture Collections for publicly available *Zygogonium* cultures but were not able to obtain any between 2015 and 2024.

### Light and fluorescence microscopy

Light‐ and fluorescence microscopy were performed using an Olympus BX41 light microscope with an attached Olympus SC30 digital camera and an Olympus IX50 inverted fluorescence microscope with an attached Olympus MicroFire S99809 digital camera (Olympus Imaging America Inc., Center Valley, PA, United States) upon receipt of the fresh samples at CSUSM. We took light microscopy images of all filaments selected for molecular analysis (see below) and additional images of *Zygogonium* filaments representing the morphological variation in each natural population. Then we analyzed a minimum of 20 filaments per population using Rincon image analysis software (Imaging Planet, Goleta, CA, United States) and measured cell width, length, width/length ratio, akinete, and aplanospore dimensions. The size ranges of *Zygogonium* filaments and spores for each natural population are presented in Table 2.

**TABLE 2:** Morphological data for the studied *Zygogonium* taxa. Legend: NR – not recorded. Note: Both samples from Ireland contain a mixture of *Z. ericetorum* and *Z. angustum* sp. nov. All other samples contain a single *Zygogonium* species.

*Zygogonium* Taxon	Phylogeny Group	DNA Reference	Field Sample	Filament width (μm)	Width/Length Ratio	Akinete characters and size (μm)	Aplanospores size (μm)	Figures
*ericetorum*	1A	JH1396, JH1397 Stancheva et al. (2014)	Tyrol, Obergurgl, Austria	15–31	0.8–4	Typical with 2 chloroplasts, elongate‐cylindrical, 19–15 × 28–52	15–23.1 × 10.1–28	Stancheva et al. (2014)
*ericetorum*	1A	1516, 1517, 1518, 1519, 1520, 1521	Tyrol, Kühtai, Austria	15.5–23.3	1–3	NR	16.7–21.5 × 17–26	4f–h; S4A–E
*ericetorum*	1A	1499, 1506	Norway 2	18.1–25.2	0.5–3	NR	20–24 × 12–25	4a–c; S3D,E
*ericetorum*	1A	1509, 1510	Norway 3	15–22.1	1.5–3.5	NR	NR	4d,e; S3E–H
*ericetorum*	1A	1477 Herburger et al. (2016)	Lochaber, Scotland	21–23	1–3.5	NR	18–22 × 18–26	Herburger et al. (2016)
*ericetorum*	1B	1479, 1480	Tyrol, Ellmau, Austria	12–23.1	0.5–3.5	Atypical with 1 or 2 chloroplasts, oval, 16–23 × 8–20	NR	5e–g; S6A–E; S8A–F
*ericetorum*	1B	1482, 1491	Ireland 4–7	14.5–30	0.5–3	Atypical with 1 or 2 chloroplasts, oval, 24–30 × 15–28	NR	5a–d; S4D,E
**angustum, sp. nov**.	**2**	**1484, 1490, 1496, 1498**	**Ireland 2–6**	**8–11.8**	**2–5.5**	**NR**	**9.6–17.1 × 11.6–23.7**	**6a–f;** **S7A,E,D,F**
*ericetorum*	3	1507, 1508	Norway 1	13.1–17.2	0.5–2	NR	13.7–16.6 × 9–21.1	7a–d; S8D–K
*ericetorum*	3	1470, 1471, 1472, 1473, 1475	Tantalus Creek, USA	14.2–19.8	1.2–3	NR	NR	7e; S8A–C

### Transmission electron microscopy

Transmission electron microscopy was performed by chemical fixation of field‐collected samples from Ireland 2–6 and Norway 1, 2, 3 as previously described (Holzinger et al., 2009). Briefly, samples were manually cleaned under the stereo microscope, fixed for 1 h in 2.5% glutaraldehyde (GA) in 10 mM cacodylate buffer (pH = 6.8), postfixed in 1% OsO_4_, dehydrated in increasing ethanol concentrations, and embedded in modified Spurr's resin. Ultrathin sections were prepared with a Reichert Ultracut (Leica Microsystems, Vienna, Austria) and counterstained with lead citrate and uranyl acetate. Sections were viewed and photographed with a Zeiss Libra 120 transmission electron microscope (TEM, Carl Zeiss AG, Oberkochen, Germany) at 80 kV, and images were taken with a TRS 2k SSCCD camera and operated by ImageSP software (Albert Tröndle Restlichtverstärker Systeme, Moorenweis, Germany).

## RESULTS

### Habitat characteristics

In Austria, *Zygogonium* was sampled from three different locations. At Obergurgl, samples were previously obtained from Mt. Schönwieskopf at 2350 m a.s.l. (Stancheva et al., 2014) from a spring pool with a temporary standing shallow water body (Figure 2a) with a pH of 4.6. Similar conditions were observed at the second Austrian sampling site at Kühtai at 2283 m a.s.l. (Figure 2b) with a pH of 6.8, which was sampled late in the growing season before snow cover (November 2015). For this location, the temperature was recorded over a year, illustrating that even in winter the temperature does not dip below −4°C due to insulating snow cover (Figure S1). At the third Austrian sampling site, Ellmau at 1150 m a.s.l., *Zygogonium* was growing in a previously described iron‐rich peat bog (Aigner et al., 2017) with a very low pH of 3.0. At this location, the abundant mats frequently become dry and are re‐submerged after heavy rain events (Figure 2c). The samples from Ireland (22 and 53 m a.s.l.) were collected in extensive peat bogs (Figure 2d) from wet soil (Ireland 2–6) or shallow puddles (Ireland 4–7) with a pH in the range from 6.6 to 7.2. *Zygogonium* samples from Norway were collected either from wet soil (N1) or from a standing shallow water body (Figure 2e, N2, 951 m a.s.l, pH = 7.6 and N3, 1106 m a.s.l., pH = 7.9). The *Zygogonium* samples collected in Scotland (45 m a.s.l., pH = 4.6) were covered by a thin water layer as previously described (Herburger et al., 2016). Samples from the United States were collected in a shallow running creek (Tantalus Creek, Figure 2f) at an elevation of 2299 m a.s.l.; the pH ranged between 3.0 and 3.5. In general, the recorded conductivities were very low and varied between 6 and 148 μS · cm^−1^, and temperatures varied between 5.3 and 18.3°C (Table 1).

### *Zygogonium* molecular phylogenetics

A complete list of available sequences is given in Table S1, but not all DNA extractions resulted in successful PCR amplification or sequence data. We used phylogenetic analyses based on sequences of the *psb*C gene, as this analysis included all samples examined in our study. Sequences of the *rbc*L gene were also available for most samples, with fewer sequences obtained for the *atp*B gene.

**FIGURE 3:**
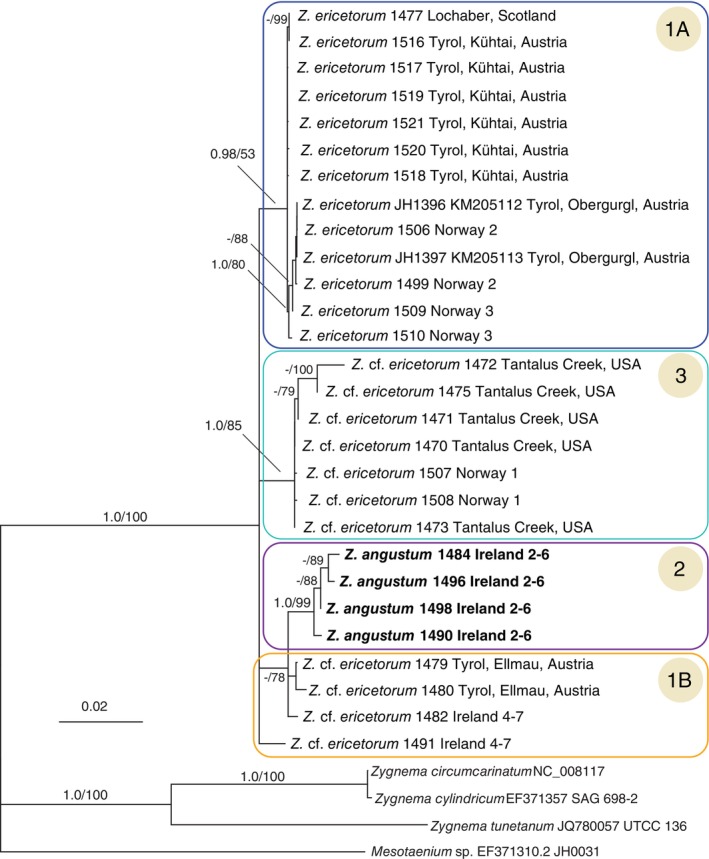
Phylogenetic relationships among strains of *Zygogonium* determined by Bayesian analysis of *psb*C gene data. The major phylogenetic groups are indicated with boxes (Groups 1A, 1B, 2, and 3). BPP values (over 0.50) plus corresponding bootstrap values (over 0.75) from a maximum likelihood analysis are shown. The scale bar = number of expected substitutions/site.

*Psb*C gene sequences were obtained from all 26 field samples, and these ranged from 707 to 1227 nucleotides (nt) in length. The *psb*C ingroup alignment contained 1228 nt, with 52 variable and 35 parsimony informative characters. Bayesian analysis of *psb*C gene data with related outgroup taxa (Figure 3) revealed a strongly supported and monophyletic *Zygogonium*. Among ingroup taxa, we distinguished three main groups, groups 1 and 3 showing the genetic variation within *Z. ericetorum*, and group 2 representing a novel species of *Zygogonium*.

Group 1 was further subdivided into group 1A and 1B. Group 1A was the largest group containing the previously described type species of the genus—*Zygogonium ericetorum* (JH 1396, 1397; Stancheva et al., 2014)—and new samples collected in Kühtai, Austria, Norway 2 and 3, and Scotland. Group 1B, in contrast, contained only samples from Ireland 4–7 and Ellmau, Austria. Group 2 contained four samples from location Ireland 2–6, described as a new *Zygogonium* sp. (see below). These samples were separated from Group 1B with high bootstrap support and differed from the others by at least seven mutations (Figure S2, network analysis). Group 3 contained samples from Norway 1 and Tantalus Creek, United States, also showing a reasonable number of mutations (at minimum three) from the rest and was also classified as *Z*. cf. *ericetorum* (Figure S2). All other samples only had 1–2 nt differences, making them quite similar in sequence.

*Rbc*L gene sequences were obtained from 15 of 26 samples, ranging from 355 to 1277 nt in length. The *rbc*L alignment was 1354 bases in length, and there were 45 variable sites, of which 27 were parsimony informative. *Rbc*L gene data could be used to support *Zygogonium* as monophyletic and sister to the Meso‐5 of Busch and Hess (2022a; Figure S3A). This data also supported Group 2, though less strongly, and there was conflicting support for other phylogenetic groups seen with *psb*C gene data (Figure 3).

*Atp*B gene data were obtained from fewer than half (11 of 26) of the field samples and from the previously studied *Zygogonium ericetorum* strain JH1397. No sequence data from group 3 samples were obtained. The sequences ranged in length from 468 to 1228 nt. The *atp*B gene alignment consisted of 1228 nt, and there were 35 variable and 23 parsimony informative characters. Analysis with outgroup taxa also showed monophyly of *Zygogonium* (Figure S3B) The Bayesian analysis of *atp*B gene data on this smaller number of ingroup specimens also produced support for morphology group 2 and group 1B as distinct from the remainder of the samples of *Z. ericetorum* in group 1A.

The concatenated three‐gene analysis included a much smaller sampling number because we restricted inclusion to those having 60 + % data occupancy. The resulting tree (Figure S3C) showed strong support for a distinct group 2 as well as intercalation of some accessions of groups 1A and 3 with group 1A.

### Morphological characterization

**FIGURE 4:**
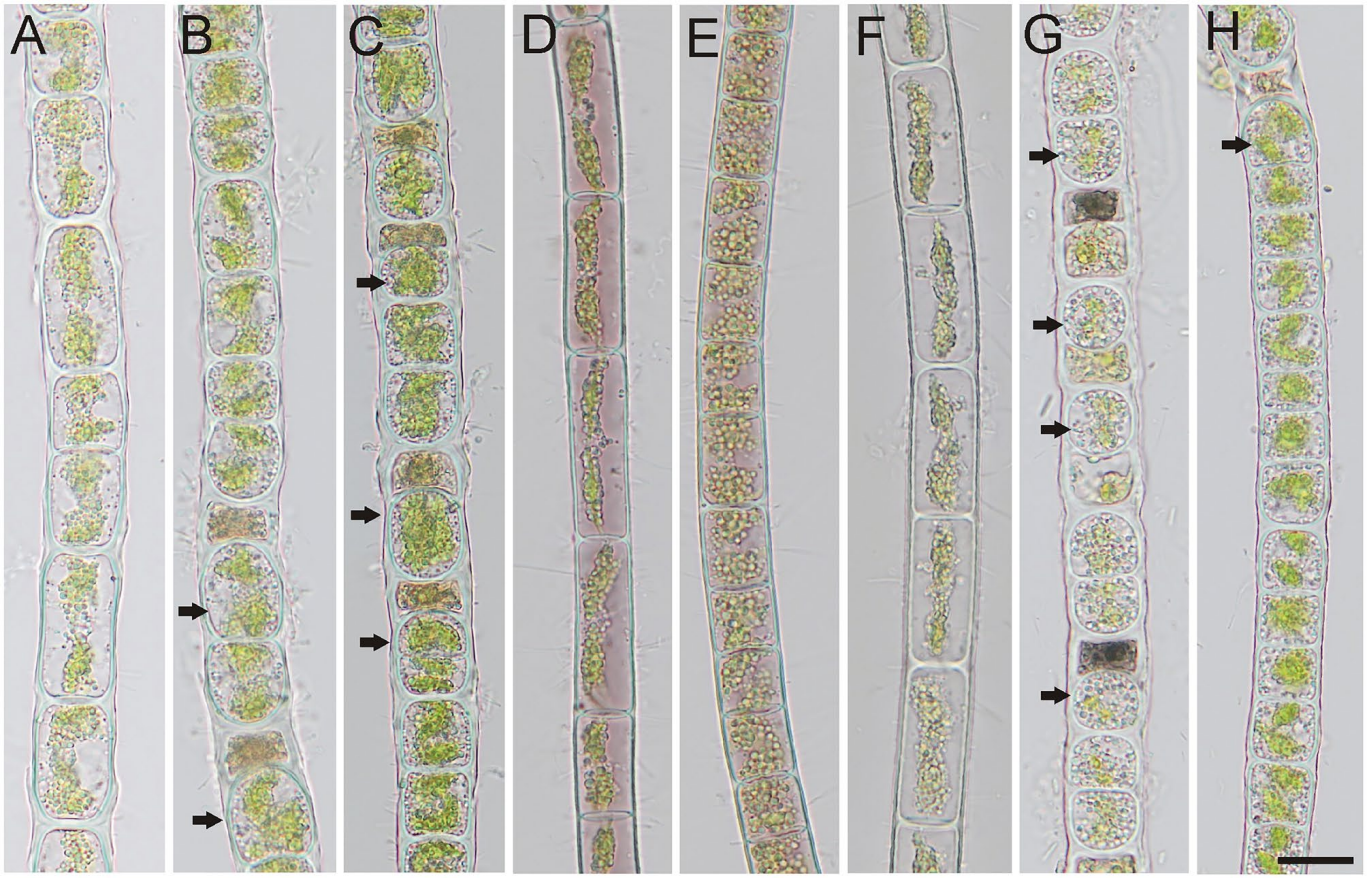
*Zygogonium ericetorum*, Group 1A. Images from Norway 2: 1499 (a) and 1506 (b, c), Norway 3: 1509 (d) and 1510 (e), Tyrol, Kühtai: 1518 (f), 1521 (g), 1519 (h); (a, d–f) vegetative filaments, (b, c, g) filaments with aplanospores (arrows), (H) filament with germinating aplanospore (arrow). Scale bar: 20 μm.

**FIGURE 5:**
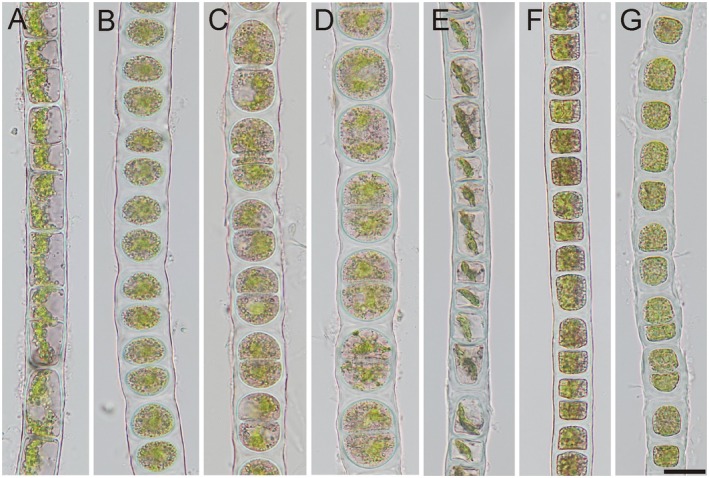
*Zygogonium* cf. *ericetorum*, Group 1B. Images from Ireland 4–7: 1482 (a, b), Ireland 4–7: 1491 (c, d), Tyrol, Ellmau: 1479 (e), Tyrol, Ellmau: 1480 (f, g); (a, e) vegetative filament, (b–d, f, g) filaments with oval akinetes with one or two chloroplasts; Scale bar: 20 μm.

**FIGURE 6:**
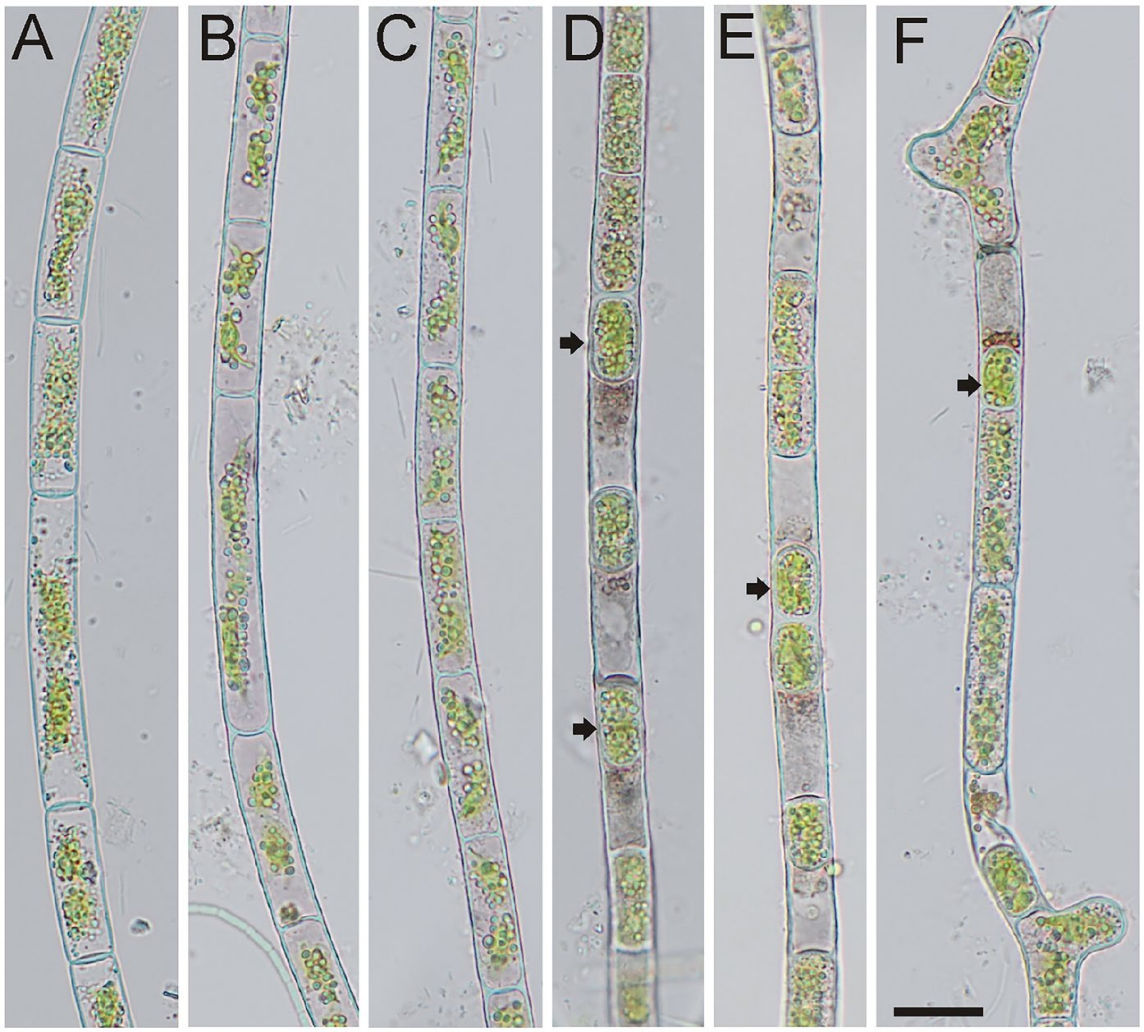
*Zygogonium angustum* sp. nov., Group 2. Images from Ireland 2–6: 1484 (a, d), 1490 (b), 1496 (c), 1498 (f); (a–c) vegetative filaments, (d–f) filaments with aplanospores (arrows); Scale bar: 20 μm.

**FIGURE 7:**
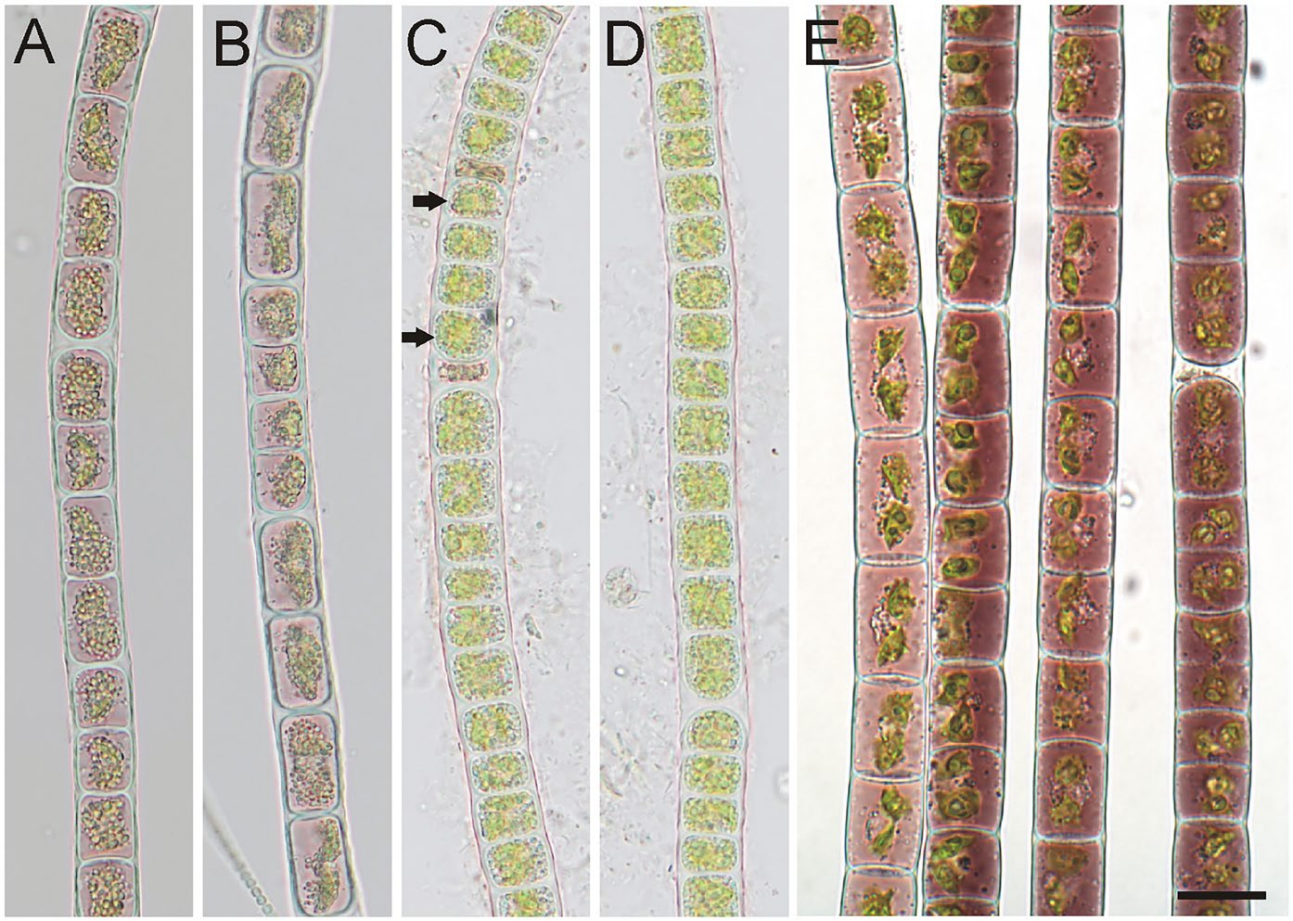
*Zygogonium* cf. *ericetorum*, Group 3. Images from Norway 1: 1507 (a, b) and 1508 (c, d), and Tantalus Creek, Yellowstone National Park, United States: 1475 (e); (a, b, e) vegetative filaments, (c) filaments with aplanospores (arrows), (d) filament with recently germinated aplanospores; (e) filaments with strong purple pigmentation. Scale bar: 20 μm.

All studied populations of *Zygogonium* showed typical vegetative filament morphology with cylindrical green and purple cells, each with two plate‐like chloroplasts with a pyrenoid (only in some akinetes, one chloroplast was observed), variable cell wall thickness, and occasional short branches (Table 2, Figures 4, 5, 6, 7, Figures S4–S9). However, there was a variation in the filament width and length/width ratio, which helped to morphologically distinguish two species. All samples contained a single *Zygogonium* species, except for the two samples from Ireland (2–6, 4–7), which were mixtures of wide and narrow *Zygogonium* filaments, corresponding to two different species. Wide filaments of *Z*. cf. *ericetorum* were sequenced from sample Ireland 4–7, while the narrow filaments of the undescribed species were sequenced from sample Ireland 2–6. Sexual reproduction was not observed in any of the samples, but aplanospores and akinete‐like cells were recorded in some populations (Table 2). The aplanospores had characteristic morphology for *Z. ericetorum* (Stancheva et al., 2014, 2016), that is, colorless, smooth walls and purple cytoplasmic residuals. However, there were significant variations in aplanospore size and outline.

The morphological grouping of *Zygogonium* populations corresponded to the subgroups according to the molecular phylogenetic data (Figure 3, Figure S3B). We observed two *Z. ericetorum* morphologies: (a) Cells with wider filaments ranging from 12 to 31 μm in diameter (see Table 2 for size ranges of each population) and occasional aplanospores with purple residue (Group 1A) or akinete‐like thick‐walled cells without residue, but no aplanospores (Group 1B, Figure 5, Figures S6 and S7) and (b) cells with narrower filaments ranging from 13 to 20 μm wide with occasional aplanospores with purple residue (Group 3, Table 2 for size ranges of each population).

The second *Zygogonium* species had narrower filaments than *Z. ericetorum* and *Z*. cf. *ericetorum* with a maximum width of 12 μm and occasionally formed aplanospores with a purple residue (Group 2).

### Group 1A: *Zygogonium ericetorum* with aplanospores (Figures S4 and S5)

This group contained *Zygogonium* genotypes from Scotland, extraction number 1477 (figures 2 and 5 in Herburger et al., 2016) and two localities in Norway (location N2), extraction numbers 1499 and 1506 (Figure 4A–C), and location N3, extraction numbers 1509 and 1510 (Figure 4D,E), which were most closely related to *Z. ericetorum* from Obergurgl, Tyrol (JH1396, JH1397, figures 2–5 in Stancheva et al., 2014) and samples from Kühtai, Tyrol, extraction numbers 1516–1521 (Figure 4f–h). Filament morphology was similar in all strains, with filament widths falling within the size range of *Z. ericetorum* (i.e., 15–31 μm, see Table 2 for size ranges of each population), variable degrees of cell wall thickness, branching, and purple content concentration. Typical aplanospores of *Z. ericetorum*, containing purple cytoplasmic residual outside the spore, were recorded in the samples from Norway 2 (Figure 4b,c), Kühtai, Tyrol (Figure 4g), and Scotland (figure 2d–g in Herburger et al., 2016). Akinetes were not recorded in this study but have previously been reported in the material from Tyrol (figure 4d in Stancheva et al., 2014).

### Group 1B: *Zygogonium* cf. *ericetorum* with akinete‐like cells (Figures S6 and S7)

This group contained two populations, Ireland 4–7 with DNA extraction numbers 1482, 1491 (Figure 5a–d) and Ellmau, Tyrol, with DNA extraction numbers 1479, 1480 (Figure 5e–g). The filament morphology was typical for *Zygogonium ericetorum*, with filament widths ranging from 12 to 30 μm (see Table 2 for size ranges of each population). However, unusual akinete‐like cells with thick, multilayered walls and no association with external purple residual were observed. These akinete‐like cells differed from the typical akinetes in *Z. ericetorum* (figure 4d in Stancheva et al., 2014) by their smaller size and almost spherical shape. The cell sap of the akinetes was either green or purple, indicating that they could also be formed in filaments lacking purple pigmentation. Some akinete‐like cells contained one chloroplast (Figures S6B–D and S7B–D). These atypical akinetes seemed to be actively dividing and probably not resting for longer periods than typical akinetes, and thus may serve different physiological purposes. Both *Zygogonium* genotypes producing atypical akinetes form subgroup 1B, within Group 1 (Figure 3). Morphological and genetic variation within *Zygogonium* subgroups 1A and 1B could be due to transitions from aquatic to terrestrial habitats, as proposed by Kirchner (1878).

### Group 2: *Zygogonium* from Ireland 2–6, with narrow filaments and aplanospores

This group contained *Zygogonium* from one location, Ireland 2–6, with the DNA extraction numbers 1484, 1490, 1496, 1498 (Figure 6a–f). The filaments and aplanospores were narrower than those in *Z. ericetorum* from group 1 and *Z*. cf. *ericetorum* from group 3 (see below). Also, the cells were at least two times longer than wide and never shorter cylindrically, as characteristic of *Z. ericetorum*. The filament widths ranged from 8 to 12 μm (see Table 2 for size ranges of each population). Given these morphological differences and the unique phylogenetic position (see above), a new species is described:

### *Zygogonium angustum* Stancheva, Lewis & Holzinger, sp. nov. (Figure S8)

#### Description

Filaments ranging from 8 to 12 μm in diameter (Figure 6a–c), sometimes forming side branches of several cells long (Figure 6F, Figure S8D–F). The cells are cylindrical, 16–73 μm long, two to six times longer than wide, with thin cell walls (Figure 6a–c). The cells are green (Figure 6a, Figure S8A–C) or have light purple cell contents (Figure 6B–F); the chloroplasts occupy only the central portion of the cell. Each cell has two plate‐like chloroplasts in close contact near the center of the cell, each with a pyrenoid. The aplanospores are elongated cylindrical and could be two to three times longer than wide. They are positioned at one end of the mother cells, which are not inflated. The aplanospore occupies one‐third to half of the volume of the mother cell and has purple cytoplasmic residual outside. The aplanospore wall is colorless and smooth (Figure 6D–F). Rarely, an aplanospore may be formed inside a branching cell (Figure S8E). Zygospores and akinetes were not observed.

#### Holotype

Specimen (UC2110196) collected from a Peat bog near Roundstone Bog Conservation Area (Derrycunlagh, Galway, Ireland) deposited in the University Herbarium at the University of California, Berkeley. Collected 1 August 2015 by Tereza Šoljaková, specimen isolated by Rosalina Stancheva.

#### Isotype

Preserved specimen fixed for TEM (# 151019), resin‐embedded material is available for reference at the Department of Botany, University of Innsbruck, Austria. Iconotype (designated here in support of the holotype): Figure 6D. Etymology: The epithet *angustum* refers to the narrow filaments, from the Latin word *angustus*—narrow.

#### Type locality

Peat bog near Roundstone Bog Conservation Area (Derrycunlagh, Galway, Ireland, 53° 27′ 16.999″ N, 9° 57′ 24.001″ W)

#### Habitat

Wet soil, pH = 7.2, conductivity 148 μS · cm^−1^.

#### Other populations examined

Ireland 4–7 (See Tables 1 and 2, Figure S8B,E)

### Group 3: *Zygogonium* cf. *ericetorum* with aplanospores (Figure S9)

This group contains *Zygogonium* from location Norway 1 with extraction numbers 1507, 1508 (Figure 7a–d) and from Tantalus Creek, Yellowstone National Park, United States, with extraction numbers 1470, 1471, 1472, 1473, 1475 (Figure 7e). The filaments were relatively narrow, ranging from 13 to 20 μm in diameter (see Table 2 for size ranges of each population). The size range was slightly below and overlapped the lower range of *Z. ericetorum*. Otherwise, no morphological differences were observed. Aplanospores and branching were recorded in Norway filaments, but not in Tantalus Creek filaments. This genotype could be a variety of *Z. ericetorum*, but data on the reproductive morphology are needed for confident descriptions.

#### Transmission electron microscopy

**FIGURE 8:**
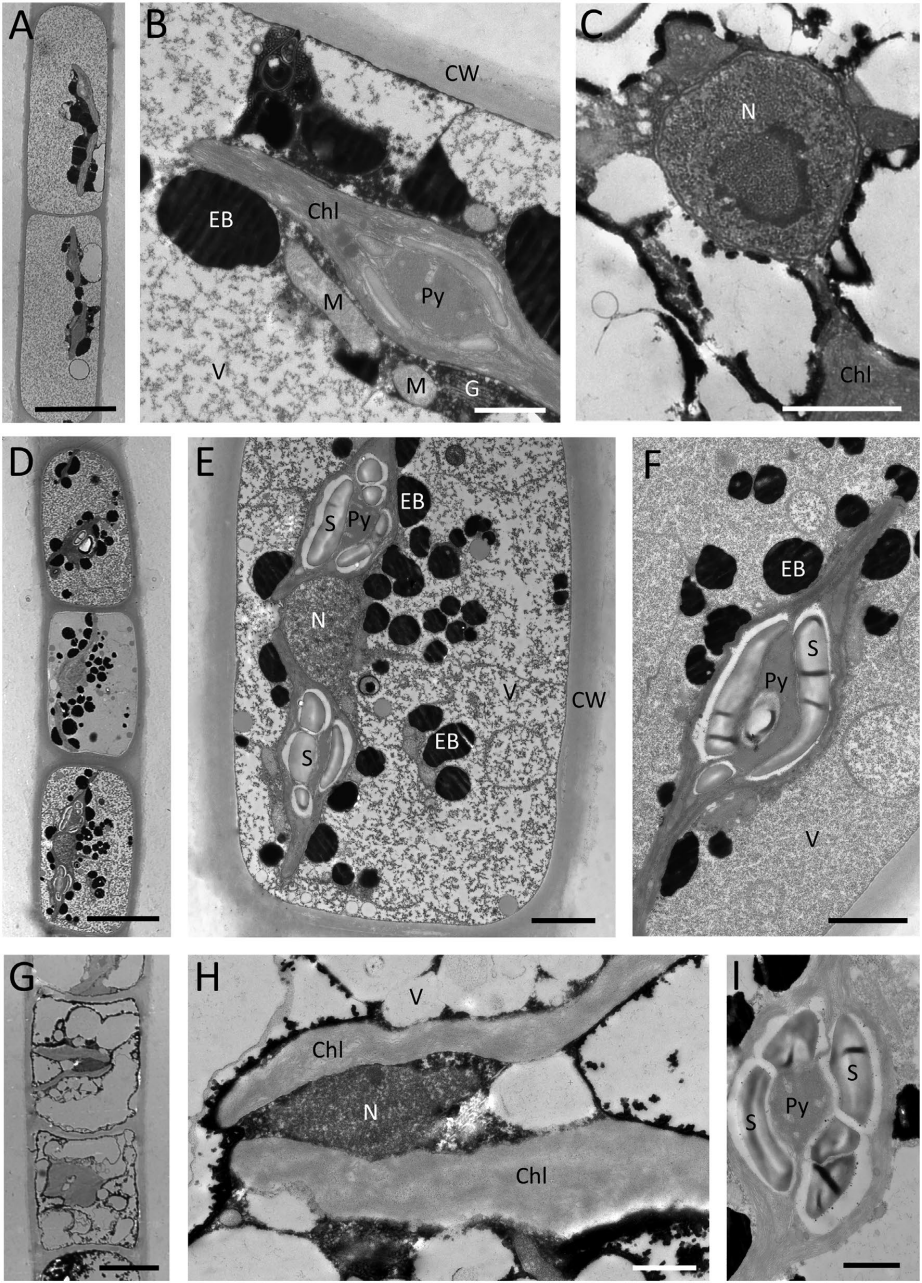
Transmission electron micrographs of Z*ygogonium angustum* sp. nov. from Ireland 2–6, Group 2 (a–c), *Zygogonium* cf. *ericetorum* from Norway 1, Group 3 (d–f) and *Zygogonium ericetorum* from Norway 3, Group 1A (g–i); (a) overview vegetative cell, (b) detail with chloroplast and pyrenoid, mitochondrion and electron dense bodies, (c) central part with nucleus, (d) overview vegetative cell, (e) detail with two chloroplasts, pyrenoids surrounded by large starch grains, nucleus and electron dense bodies, (f) detail chloroplast with pyrenoid, (g) overview, (h) central part with nucleus and two chloroplasts, (i) detail of pyrenoid with starch grains. Chl, chloroplast; EB, electron dense body; M, mitochondrion; N, nucleus; Py, pyrenoid; S, starch; V, vacuole; Scale bars: a, d, g 10 μm, b–c, e–f, h–i 1 μm.

In the present study, TEM was performed for all samples collected in Norway and samples collected in Ireland 2–6 (Figure 8). Vegetative filaments from Ireland 2–6 (phylogenetic group 2) described as *Zyogonium angustum* sp. nov. showed narrow filaments containing small chloroplasts with pyrenoids surrounded by flat and small starch grains (Figure 8a–c). Moreover, these filaments had very thin and smooth cell walls when compared to samples from Norway 1 (group 3, Figure 8d–f) and Norway 3 (group 1A, Figure 8g–i). The chloroplasts were plate‐like in all investigated samples and contained a pillow‐shaped pyrenoid, surrounded by starch grains (Figure 8). The nucleus was positioned in the cell center, and the vacuoles mostly had a granulated content. Samples from location Norway 1 (group 3) also had two plate‐like chloroplasts (Figure 8d,e); the pyrenoids were surrounded by large starch grains (Figure 8e,f). Numerous electron‐dense bodies surrounded the chloroplasts (Figure 8d–f). Samples from Norway 3 (phylogenetic group 1A) had small plate‐like chloroplasts, fewer electron‐dense bodies (Figure 8g,h) and the pyrenoids were surrounded by starch grains (Figure 8i). Overall, transmission electron microscopy confirmed the typical features of *Zygogonium* ultrastructure.

## DISCUSSION

In this study, we have provided an integrative molecular, morphological, ultrastructural, and ecological characterization of *Zygogonium* populations from different locations in Europe (Austria, Scotland, Ireland, Norway) and Yellowstone National Park in the United States. The overall genetic variability was demonstrated with three main groups, with one group often subdivided into two subgroups (group 1A, 1B). The phylogenetic groups indicated in Figure 3 corresponded to distinct morphological characteristics of the filaments and spores. Analyses of data from the three genes individually and in a concatenated analysis provided strong support for the distinction of group 2, but the signal in the *rbc*L gene was quite low. For the other groups, we detected conflict among topologies from different genes, in particular, the placement of *Z*. cf. *ericetorum* 1491 and the placement of group 3, which were sometimes within group 1. One explanation for this pattern could be low variation, such as in the *rbc*L gene, or the lack of comparable data across all accessions (group 3 lacked *atp*B gene data, and different samples were present across analyses). Another possible explanation is that these entities may represent incipient lineages of *Z. ericetorum* but with insufficient time to separate. With additional information, we may have sufficient evidence to recognize these as new species. We consider group 1A to represent *Z. ericetorum*, group 2 to represent *Z. angustum*, and the remaining groups have been designated as *Z*. cf. *ericetorum*, as they may be assigned to that species but may also represent distinct taxa.

### Phylogenetic analysis shows genetic variability

Most of the phylogenetic groups contained samples from different geographic origins, suggesting a widespread distribution of these molecularly distinct lineages. For example, group 1A samples originated from Austria (Obergurgl, Kühtai), Scotland, and two sites in Norway. This group also contained the previously characterized and sequenced strain representing the type species *Zygogonium ericetorum* from Tyrol, Austria (Stancheva et al., 2014). Group 1B contained samples from Austria (Ellmau) and Ireland, and group 3 samples contained samples from Norway and the United States. Group 2 contained samples only from one location in Ireland. Therefore, we analyzed if distinct morphological characteristics could be observed for the individual phylogenetic groups. There were only a limited number of morphological features to be indicative for species determination in *Zygogonium*, as previously described (Stancheva et al., 2014), such as filament width and morphology of reproductive cells. Even the conjugation features in *Zygogonium* did not provide structural diversity, which could inform the species identification. In *Zygogonium*, the zygospores are always formed in the conjugation canal and do not have any specific color and structure of the walls, in contrast to *Zygnema* species that may have zygospores positioned either in the conjugation canal or in the cells, and the mesospore has numerous variations in color and ornamentation (Stancheva et al., 2012; Transeau, 1951). We largely relied on filament morphology for species identifications, but this may be misleading because the formation of rhizoids, thickened walls, and cell length vary with environmental conditions and cell growth and division.

### Vegetative morphology shows a large variation in cell width

*Zygogonium ericetorum* vegetative cells contained two plate‐like irregularly shaped chloroplasts, each with a central pyrenoid (Stancheva et al., 2014), which was confirmed by light‐ and transmission electron microscopy (see below) in the present study.

The cell width of *Zygogonium ericetorum* may vary from 12 to 31 μm (Kadlubowska, 1984; Rundina, 1998; Stancheva et al., 2014; Transeau, 1951), a range that was also observed in *Z. ericetorum* group 1 in this study. *Zygogonium* cf. *ericetorum* group 3 samples from location Norway 1 and Tantalus Creek in the United States, with an intermediate filament width of 13–20 μm, were observed distinctly in the phylogenetic analysis, with at least three mutations (Figure S2); however, their morphological appearance and the overlapping cell widths with *Z. ericetorum* did not provide support for its separation as a novel species. Furthermore, we did not observe reproductive cells in group 3, which are important for species delineation. We also recorded variation in the cell wall thickness and layering, often associated with color variation from green to light or deep purple within most *Zygogonium* populations studied. The cell wall thickness in *Z*. *ericetorum* varies strongly with cell age and according to the environmental conditions the algae have been exposed to (e.g., Fritsch, 1916; Fritsch & Haines, 1923; Holzinger et al., 2010; Hoppert et al., 2004). Only the filaments from Tantalus Creek and Norway 3 had thin cell walls and predominantly purple cells, but this could be due to undersampling.

The most striking difference in the *Zygogonium* samples of this study was observed in the phylogenetic analysis of group 2 with the occurrence of particularly thin filaments, with widths of only 8–12 μm. Filaments with this morphology were recorded only in the samples from Ireland (location 2–6) and used for DNA extractions. The narrow filament width below the lower limit of the cell width of *Z. ericetorum*, together with the molecular differences (network‐based analysis of the *psb*C gene sequences showed at least seven mutations, Figure S2, network‐based analysis) gives strong support for the description of a new species of *Zygogonium*. There are several *Zygogonium* species described in the previous literature with narrow filaments (e.g., *Z. tenue*; Kützing, 1849). The newly described species *Z. angustum* sp. nov. is similar in regard to filament width to *Z. pectosum*, described from wet seepage slopes in Louisiana, United States. However, *Z. pectosum* differs because it has shorter aplanospores with slate‐blue walls and a sporangial wall with a 2–4 μm thick layer of pectic compounds (Taft, 1944; Transeau, 1951). Also, *Z. punctatum*, collected in De Ridder, Louisiana, United States, on roadside seepage slopes, was described with small filaments (9–12 μm × 30–45 μm) containing small irregular globose chloroplasts, but no information on aplanospores was given (Taft, 1944). The zygospores of this species were described as globose to subglobose with a yellow to yellow‐brown mesospore (Taft, 1944); however, in the present study, we did not observe sexual reproduction. Another species with narrow filaments is *Z. hansgirgii*, but aplanospores were described with a brown median wall, with small angular protuberances (verrucose; Transeau, 1933), which we did not observe. Furthermore, we cannot genetically compare *Z. angustum* sp. nov. with other species because, at this point, molecular data exist only for *Z. ericetorum* (Stancheva et al., 2014).

Transmission electron microscopy of vegetative cells illustrated the typical *Zygogonium* ultrastructure as previously shown in *Z. ericetorum* from Obergurgl, Austria (Aigner et al., 2013; Holzinger et al., 2010), and Scotland (Herburger et al., 2016). Previously published *Z. ericetorum* ultrastructures showed thick multilayered cell walls (Aigner et al., 2013; Herburger et al., 2016; Holzinger et al., 2010; Hoppert et al., 2004). Thus, the chloroplasts of *Zygogonium* are clearly distinct from the star‐shaped chloroplasts in *Zygnema*, as previously discussed (Stancheva et al., 2014). Moreover, the chloroplasts were surrounded by numerous electron‐dense bodies, as previously reported (Aigner et al., 2013).

### Aplanospores in *Zygogonium*

The aplanospores documented in the present study from group 1A and group 3 (Figure 4b,c,g; Figure 7c) were similar to the previously described aplanospores of *Zygogonium ericetorum* from the collection site Obergurgl, Tyrol (Stancheva et al., 2014). The aplanospores of *Z. ericetorum, Z*. cf. *ericetorum*, and *Z. angustum* had similarly colorless walls and unilateral positions of the purple cytoplasmic residue. The only exception was *Z. ericetorum* from Scotland (Herburger et al., 2016), included in group 1A, which had aplanospores positioned in the middle of the mother cells, resulting in the cytoplasmic residuals at both sides of the newly developed aplanospore (see figure 2f–g in Herburger et al., 2016; described as an “encysted” cell). It may be noteworthy that this aplanospore position was already illustrated by West and Starkey (1915, figure 4D), but without further explanations. It remains unclear if this morphological difference in the appearance of the aplanospore of *Z. ericetorum* compared to that described by Stancheva et al. (2014) has a taxonomic relevance. The results of the phylogenetic analysis place this sample closely related to a *Z. ericetorum* sample from the location Kühtai in Tyrol, Austria.

### Akinetes in *Zygogonium*

When vegetative *Zygogonium* cells are exposed to natural desiccation, akinetes with thick, multilayered cell walls are formed (Fritsch, 1916). Typical akinetes of *Z. ericetorum* were reported in Stancheva et al. (2014), with thick cell walls and an elongated elliptical shape following the outline of the cell. However, occasionally, rounded cells with massive cell walls have been reported in *Zygogonium* (e.g., Hoppert et al., 2004). In the present study, we were able to illustrate unusual oval akinetes that had a nearly spherical cell shape in samples from Ireland (observed in both investigated localities but only sequenced and illustrated from location 4–7). Akinetes with a cell lumen not as spherical and with a more rectangular appearance were observed in samples from Ellmau, Tyrol, Austria (Figure 5f,g). Akinetes often contain high concentrations of lipid droplets, predominantly in the cell periphery (Figures 5b–d,f,g and 6; Figures S5F,G, S6B,C, and S7B–E), Interestingly, phylogenetic group 1B contained only these two samples, possibly suggesting a phylogenetic significance. This process is a quick mode of cellular protection as it does not require complicated morphological transformations characteristic of the aplanospores, which were not observed in the filaments containing akinetes in both localities. The fact that the akinetes have either green or purple content indicates that they could be formed in filaments in different physiological states. The shape of the cells could be a result of the growth conditions; after cell division, the young cells are half the length of the mother cell. Under favorable conditions, the outer cell wall elongates and expands until the cells reach their full length. When conditions are changing, for example due to a desiccation event, they might not be elongating but instead quickly forming protective multilayered cell walls, resulting in this spherical appearance of the akinetes. When moisturized again (by rainfall, for example), they might start dividing again, as observed in samples from Ireland 4–7 (Figure 5c,d) that had these spherical akinetes with young cross walls resulting from a recent cell division. Due to the fast cell division associated with akinete formation and germination, some cells may contain a single chloroplast, as previously reported in *Z. ericetorum* (figure 2 in Fritsch, 1916, West & Fritsch, 1927) and confirmed in this study by fluorescence microscopy (Figure S6C,D). Gau (1934) described similar filament morphology in *Z. ericetorum* as “pearl‐ribbon like appearance (Type 5, p. 13)” of the filaments with cell lumen round and filled with storage compounds as results of nutrient starvation and water loss in laboratory experiments. This “pearl‐ribbon like appearance,” especially in the samples from Ireland is so unusual and little‐known as a characteristic of *Zygogonium* that it has resulted in misidentifications (e.g., figure 12G in John & Williamson, 2009, named similar filament from Ireland *Microspora irregularis*).

### Ecological remarks

In this study, we collected *Zygogonium* green and purple filaments from various permanent or ephemeral freshwater habitats with shallow standing or running waters, damp soil, and peat. The localities spanned from lowland blanket bogs in Ireland to mountain wetlands in Tyrol at an altitude of 2350 m a.s.l. Water was acidic to slightly alkaline (pH 4.6–7.6) with extremely acidic conditions (pH 3–3.5) recorded in Tantalus Creek in Yellowstone National Park, United States, and Ellmau, Tyrol, Austria. This study was not designed to investigate the ecology of the *Zygogonium* populations; however, when available, we have included measurements of the pH values and the conductivity of the sampling sites (Table 1). The habitats in which *Z. ericetorum* typically occur exhibit rather low conductivity with a maximum of 148 μS · cm^−1^ in Ireland 2–6 as expected for oligotrophic sites (Pichrtová et al., 2018). It is generally believed that *Z. ericetorum* grows best on acidic soils (e.g., Herburger et al., 2016; Kleeberg et al., 2006; Lynn & Brock, 1969), and this holds true for most of the sampling sites in the present study; however, sometimes neutral to slightly alkaline pH values were reported from Norway and Ireland (Table 1). This might reflect the broad tolerance of *Z. ericetorum* to various conditions, as already pointed out in very early studies (West & Starkey, 1915). Also, in the present study, the samples were collected from totally submerged sampling sites (e.g., in Norway 2, 3) as well as more terrestrial sampling sites in Ellmau, Austria, or Scotland (Herburger et al., 2016). It is interesting to note that chytrid parasites, which are common in Zygnematacean algae, were observed in some of the studied filaments, and illustrated in both *Z. ericetorum* (Figure S10A–E) and *Z. angustum* sp. nov. (Figure S8E).

Ecological studies of *Zygogonium* deserved more attention, but they are limited by the rare distribution of this organism in remote areas, often high mountains or protected national parks, such as Yellowstone, which was included in our study. Furthermore, difficulties in establishing cultures of this organism, which is sensitive to artificial conditions, complicate its investigations. Shipping live material of *Zygogonium* between continents and four laboratories in Europe and the United States presented challenges for and limitations to our study but allowed us to reveal substantial genetic and morphological variation within its natural populations. More research is needed to improve our knowledge about the phenotypic and genotypic variation in this desiccation‐ and UV‐tolerant alga in conjunction with its ecological plasticity. Fortunately, there has been recent progress in cultivating *Ancylonema alaskanum* (Remias & Procházková, 2023), giving good prospects that this will soon also be possible with the closely related *Zygogonium*. Such laboratory‐grown cultures will greatly enhance our understanding of these algae by facilitating more detailed phylogenetic analysis as well as enabling the combination of field research with experimental approaches.

## CONCLUSIONS

Summarizing our results from *Zygogonium* samples from locations in Austria, Ireland, Scotland, Norway, and the United States, we have shown genetic variability in the investigated samples by sequences of the *psb*C gene, and these formed distinct phylogenetic groups. The genetic variability is also reflected in the morphology of the vegetative filaments. Although many samples contained aplanospores, akinetes were reported only in samples from Ellmau, Tyrol, Austria, and Ireland in the present study. Most samples contained filaments belonging to *Z. ericetorum* or *Z*. cf. *ericetorum*, which showed typical vegetative morphology with variation in cell width but phylogenetically clustered together in group 1. The *Zygogonium* cluster was further phylogenetically subdivided into two subgroups characterized by either aplanospores with cytoplasmic residue outside the aplanospore or unusual round akinetes, surrounded by thick multilayered cell walls. Group 2 contained very narrow filaments (up to 12 μm) that were distinct from *Z. ericetorum* and has been described as a new species *Zygogonium angustum* sp. nov. in the present study, based on morphological as well as genetic observations. In contrast, group 3, with slightly narrower filaments up to 20 μm may be a variant of the typical *Z. ericetorum*.

In the present study, it was not possible to establish *Zygogonium* cultures, which might be have been due to the selected growth medium, and future studies will have to test systematically which media are sufficient. Moreover, in future studies, more sampling sites should be included, and even more importantly, the morphologies observed at one sampling site have to be examined very carefully.

## AUTHOR CONTRIBUTIONS

**Rosalina Stancheva:** Conceptualization (equal); formal analysis (equal); investigation (equal); methodology (equal); visualization (equal); writing – original draft (equal). **Louise A. Lewis:** Data curation (equal); formal analysis (equal); investigation (equal); methodology (equal); resources (equal); writing – review and editing (equal). **John Hall:** Data curation (equal); investigation (equal); methodology (equal); writing – review and editing (supporting). **Tereza Šoljaková:** Investigation (equal); methodology (equal); writing – review and editing (supporting). **Charlotte Permann:** Formal analysis (equal); investigation (equal); visualization (supporting); writing – review and editing (equal). **Andreas Holzinger:** Conceptualization (lead); formal analysis (equal); funding acquisition (lead); investigation (equal); methodology (equal); project administration (lead); supervision (lead); writing – original draft (equal).

## Supporting information


**Figure S1.** Temperature course monitored over the season 2016/2017 at the location “Kühtai” (47.222333, 11.027133); when the temperature is not fluctuating in a daily course, the site was snow covered (i.e., early November 2016—mid May 2017).
**Figure S2.** Network‐based sequence analysis of the *psb*C gene. Note that networks can be joined in odd ways showing connections between tips. The size of the ball indicates the number of strains with that same haplotype. The hatch marks show mutations separating haplotypes. Some haplotypes are intermediate but were not observed (Labels absent).
**Figure S3.** Composite figure of trees resulting from Bayesian analysis of ingroup plus outgroup taxa (A) *rbc*L tree (only ingroup taxa shown due to long outgroup branch lengths) (B) *atp*B tree (only ingroup taxa shown due to long outgroup branch lengths) and (C) concatenated 3‐gene analysis for isolates having 60 + % data occupancy, in which certain branches leading to outgroup taxa are half scale (indicated by two parallel lines). In all analyses *Zygogonium* is monophyletic. Morphology groups within *Zygogonium* as detailed in Table 1–2 and supported by *psb*C data are shown in colored boxes. In all cases group 2 is monophyletic, but other groups sharing morphological features are not. BPP values are shown on branches of the tree. Scale bars = expected number of substitutions/site.
**Figure S4.** *Zygogonium ericetorum* Group 1A. Filaments from Norway 2 (A‐D) and Norway 3 (E‐H); (A, B, E‐H) vegetative filaments, (C, D) filaments with germinating aplanospores (arrows); Scale bar: 20 μm.
**Figure S5.** *Zygogonium ericetorum* Group 1A filaments from Tyrol, Kühtai (A‐C) and Group 1B filaments from Ireland 4–7 (D‐G); (A, B, D, E) vegetative filaments, (C) filament with aplanospores (arrow), (F) filament with oval akinetes with one or two chloroplasts, (G) filament with germinating akinetes; Scale bar: 20 μm.
**Figure S6.** *Zygogonium* cf. *ericetorum* Group 1B filaments from Ireland 2–6 (not sequenced); (A) vegetative filament, (B–D) filaments with oval akinetes with one or two chloroplasts (arrows show akinetes with single chloroplast). Images C and are the same filament—image D obtained by fluorescence microscopy; Scale bar: 20 μm.
**Figure S7.** *Zygogonium* cf. *ericetorum* Group 1B filaments from Tyrol, Ellmau (A‐E); (A, E) vegetative filaments, (B–D) filaments with oval akinetes with one or two chloroplasts, (E) filament with side branch; Scale bar: 20 μm.
**Figure S8.** *Zygogonium angustum*, sp. nov. Group 2 filaments from Ireland 2–6 (A, C, D, F) and Ireland 4–7 (B, E ‐ not sequenced); (A‐C, D, F) vegetative filaments, (E) filament with an aplanospore inside the branching cell (black arrow) and parasite cyst inside another cell (white arrow), (D–F) filament with side branches; scale bar: 20 μm.
**Figure S9.** *Zygogonium* cf. *ericetorum* Group 3 filaments from Tantalus Creek in Yellowstone National Park, United States (A‐C) and Norway 1 (D‐K); (A – F, K) vegetative filaments, (G‐J) filaments with aplanospores (arrows), (I‐K) filament with side branches, note the formation of aplanospore inside the branching cell (I arrow); Scale bar: 20 μm.
**Figure S10.** Fungal parasite on *Zygogonium* cf. *ericetorum* Group 1B from Ellmau, Tyrol; (A‐C) extracellular sporangium of chytrid parasite (black arrows) on the top of *Zygogonium* filaments, (C‐E) Intracellular parasite cysts (white arrows) inside *Zygogonium* cells missing chloroplasts; Images obtained by differential interference contrast light microscopy; Scale bar: 20 μm.

**Table S1.** Sequence data availability of the *Zygogonium* collections used in this study and of outgroup taxa for phylogenetic analyses. DNA numbers correspond to the DNA number in Tables 1–2, with JH corresponding to collections by John Hall. NCBI accession numbers for the new sequences are shown in boldface font, and these are followed by the length of the determined sequence in nt, shown in parenthesis. ND = sequence not determined during our study; n/a = published sequence not available.

## Data Availability

The newly generated sequences were deposited in Genbank under accession numbers summarized in Table S1.
