# Supplementary material for: Genetic and morphological variation in the genus Zygogonium (Zygnematophyceae, Charophyta) from localities in Europe and North America and description of Z. angustum, sp. nov
Source: J Phycol. 2025 Apr 9;61(3):587–606. doi: 10.1111/jpy.70012 (PMC12168101; doi:10.1111/jpy.70012)
Supplement: Supplementary file 1 — Figure S1. Temperature course monitored over the season 2016/2017 at the location “Kühtai” (47.222333, 11.027133); when the temperature is not fluctuating in a daily course, the site was snow covered (i.e., early November 2016—mid May 2017). Figure S2. Network‐based sequence analysis of the psbC gene. Note that networks can be joined in odd ways showing connections between tips. The size of the ball indicates the number of strains with that same haplotype. The hatch marks show mutations separating haplotypes. Some haplotypes are intermediate but were not observed (Labels absent). Figure S3. Composite figure of trees resulting from Bayesian analysis of ingroup plus outgroup taxa (A) rbcL tree (only ingroup taxa shown due to long outgroup branch lengths) (B) atpB tree (only ingroup taxa shown due to long outgroup branch lengths) and (C) concatenated 3‐gene analysis for isolates having 60 + % data occupancy, in which certain branches leading to outgroup taxa are half scale (indicated by two parallel lines). In all analyses Zygogonium is monophyletic. Morphology groups within Zygogonium as detailed in Table 1–2 and supported by psbC data are shown in colored boxes. In all cases group 2 is monophyletic, but other groups sharing morphological features are not. BPP values are shown on branches of the tree. Scale bars = expected number of substitutions/site. Figure S4. Zygogonium ericetorum Group 1A. Filaments from Norway 2 (A‐D) and Norway 3 (E‐H); (A, B, E‐H) vegetative filaments, (C, D) filaments with germinating aplanospores (arrows); Scale bar: 20 μm. Figure S5. Zygogonium ericetorum Group 1A filaments from Tyrol, Kühtai (A‐C) and Group 1B filaments from Ireland 4–7 (D‐G); (A, B, D, E) vegetative filaments, (C) filament with aplanospores (arrow), (F) filament with oval akinetes with one or two chloroplasts, (G) filament with germinating akinetes; Scale bar: 20 μm. Figure S6. Zygogonium cf. ericetorum Group 1B filaments from Ireland 2–6 (not sequenced); (A) vegetative f [file JPY-61-587-s002.pdf]

Supplementary Figures:

Genetic and morphological variation in the genus *Zygogonium* (Zygnematophyceae, Charophyta) from localities in Europe and North America and description of a new species *Z. angustum*, sp. nov.

Journal of Phycology

Rosalina Stancheva[1], Louise A. Lewis[2], John Hall[3], Tereza Šoljaková[4], Charlotte Permann[5], Andreas Holzinger[5*]

*Author for correspondence: Andreas.Holzinger@uibk.ac.at

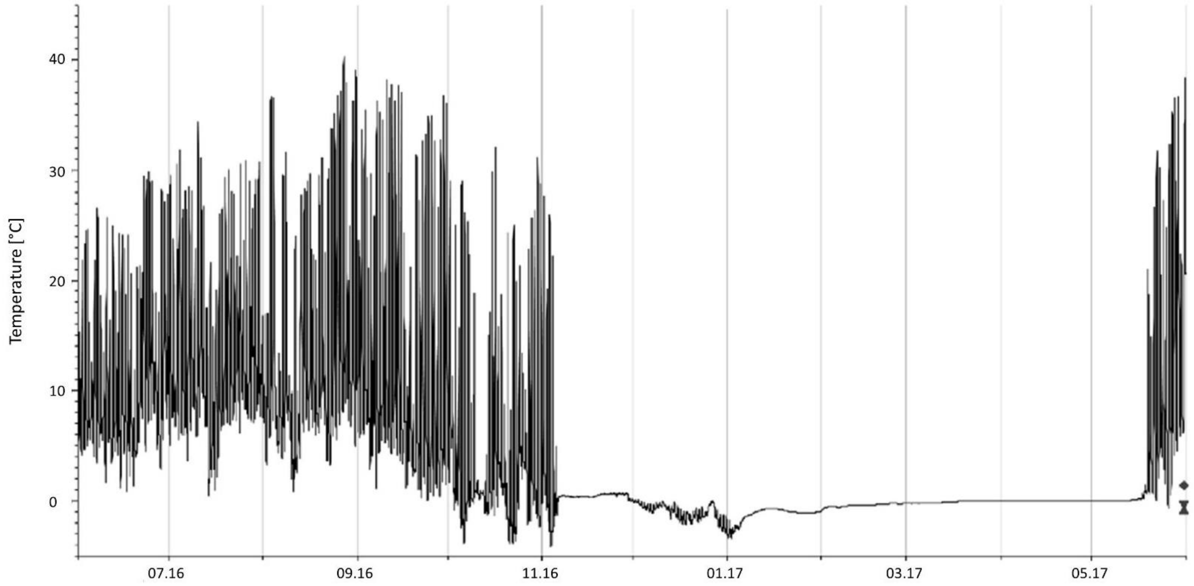

Figure S1 Temperature course monitored over the season 2016/2017 at the location 'Kühtai' (47.222333 11.027133); when the temperature is not fluctuating in a daily course, the site was snow covered (i.e. early November 2016 – mid May 2017.

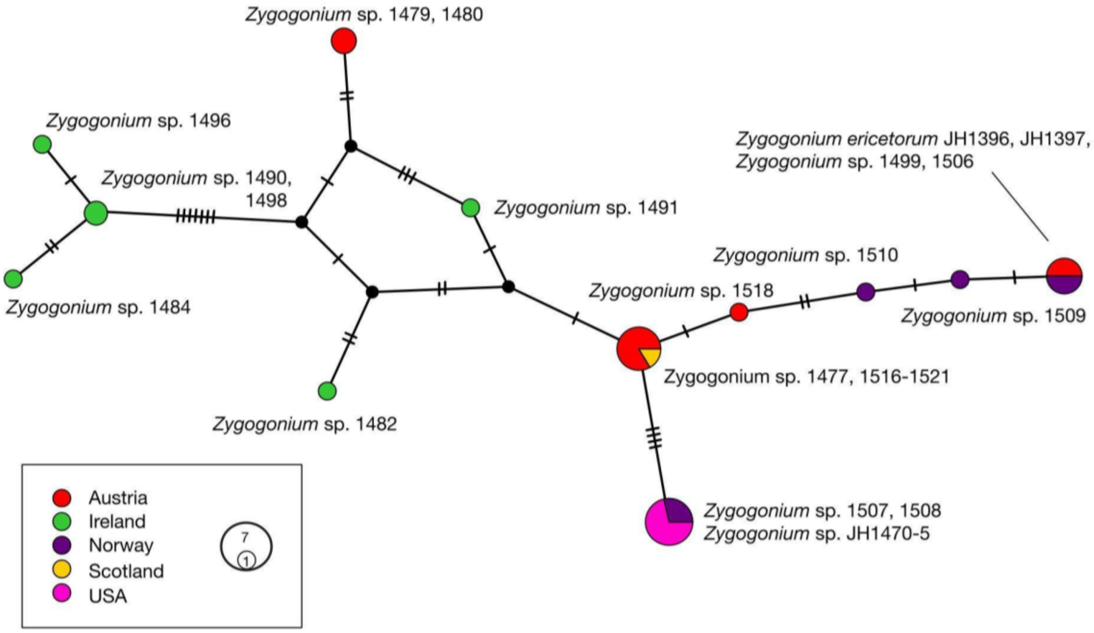

Figure S2 Network based sequence analysis of the *psbC* gene. Note that networks can be joined in odd ways showing connections between tips. The size of the ball indicates the number of strains with that same haplotype. The hatch marks show mutations separating haplotypes. Some haplotypes are intermediate but were not observed (Labels absent).

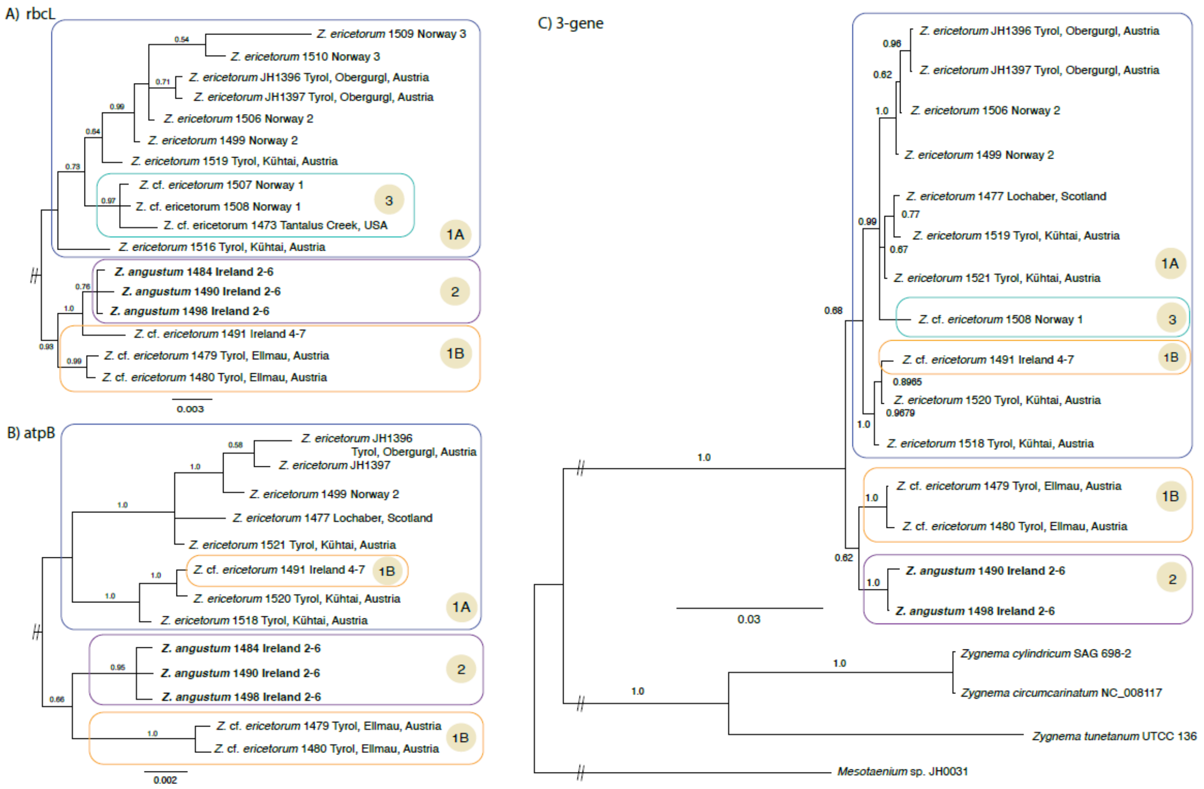

Figure S3

Composite figure of trees resulting from Bayesian analysis of ingroup plus outgroup taxa (A) *rbc*L tree (only ingroup taxa shown due to long outgroup branch lengths) (B) *atp*B tree (only ingroup taxa shown due to long outgroup branch lengths) and (C) concatenated 3-gene analysis for isolates having 60+% data occupancy, in which certain branches leading to outgroup taxa are half scale (indicated by two parallel lines). In all analyses *Zygogonium* is monophyletic. Morphology groups within *Zygogonium* as detailed in Table 1-2 and supported by *psb*C data are shown in colored boxes. In all cases group 2 is monophyletic, but other groups sharing morphological features are not. BPP values are shown on branches of the tree. Scale bars = expected number of substitutions/site.

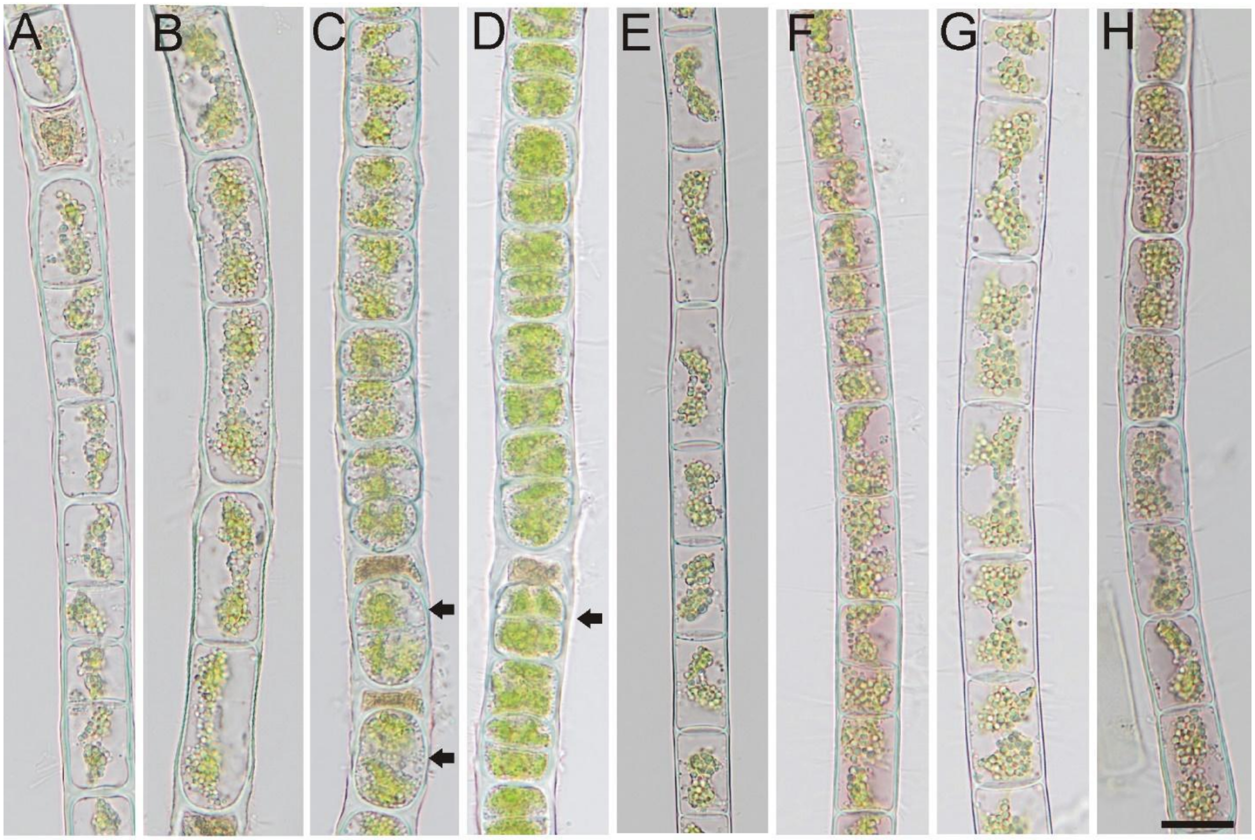

Figure S4 *Zygogonium ericetorum* Group 1A. Filaments from Norway 2 **(A-D)** and Norway 3 **(E-H)**; (A, B, E-H) vegetative filaments, (C, D) filaments with germinating aplanospores (arrows); Scale bar: 20 µm.

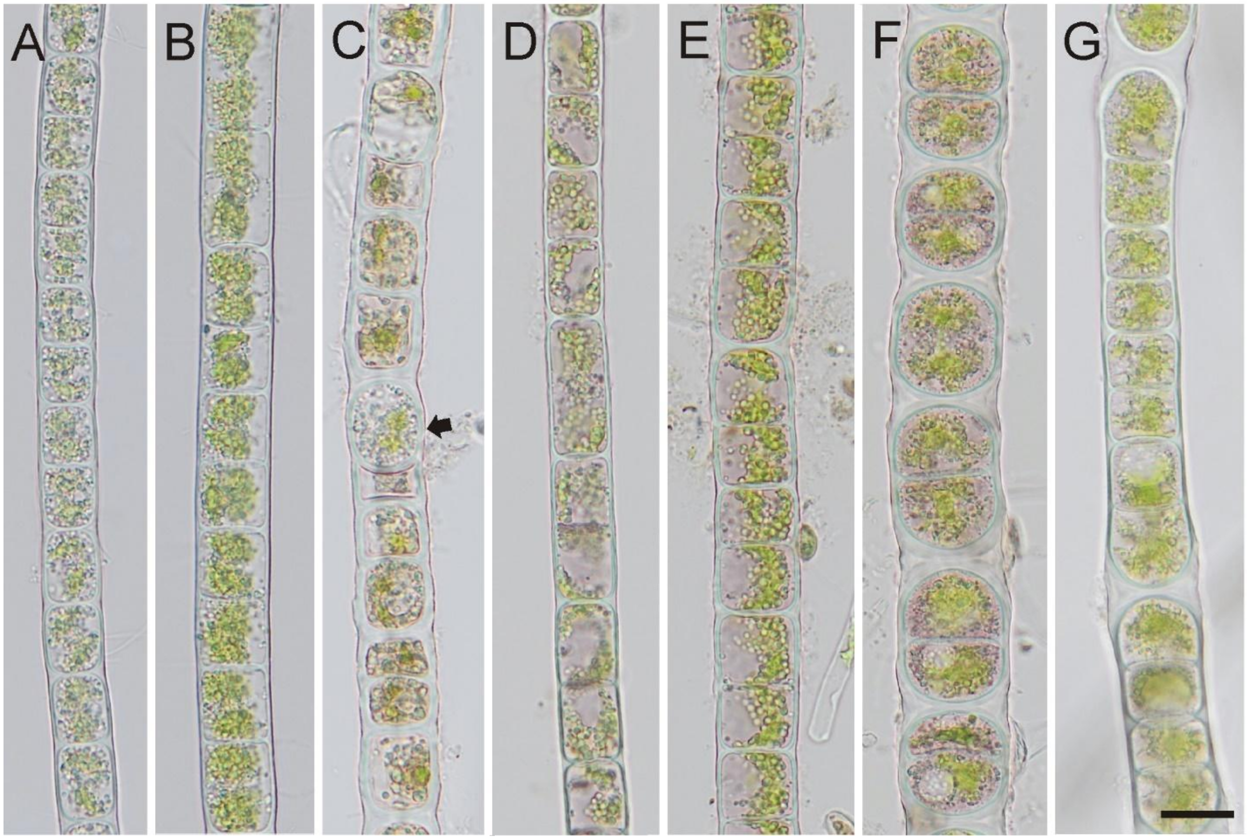

Figure S5 *Zygogonium ericetorum* Group 1A filaments from Tyrol, Kühtai **(A-C)** and Group 1B filaments from Ireland 4-7 **(D-G)**; (A, B, D, E) vegetative filaments, (C) filament with aplanospores (arrow), (F) filament with oval akinetes with one or two chloroplasts, (G) filament with germinating akinetes; Scale bar: 20 µm.

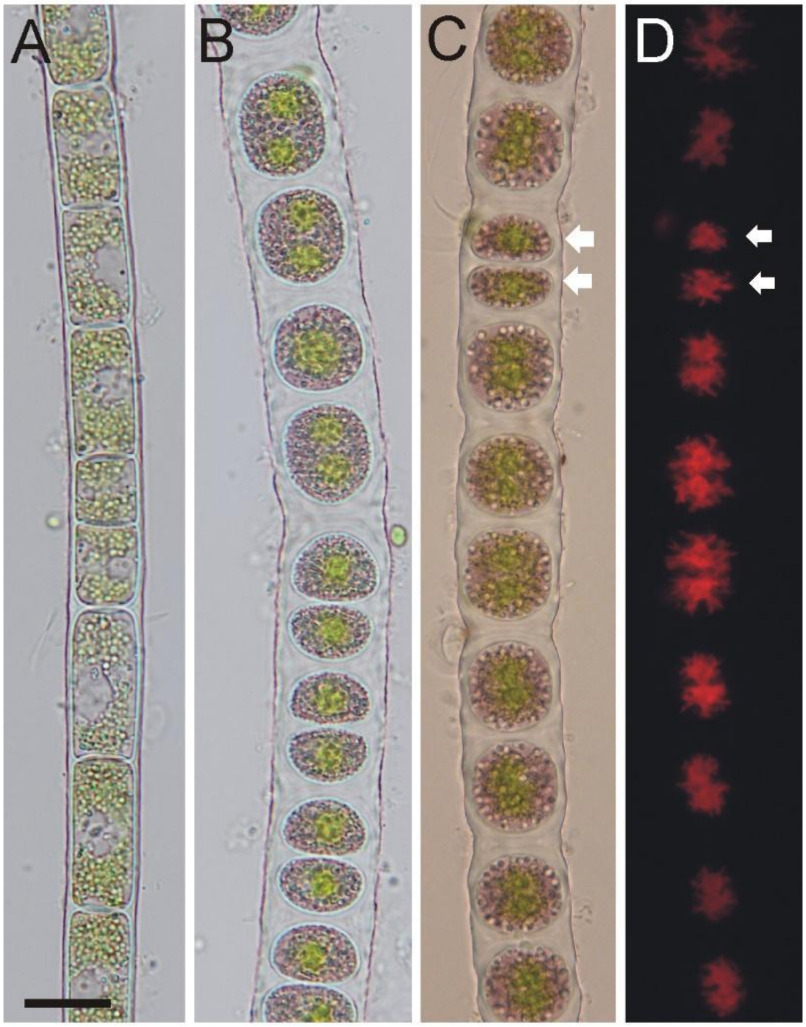

Figure S6 *Zygogonium* cf. *ericetorum* Group 1B filaments from Ireland 2-6 (not sequenced); (A) vegetative filament, (B – D) filaments with oval akinetes with one or two chloroplasts (arrows show akinetes with single chloroplast). Images C and are the same filament - image D obtained by fluorescence microscopy; Scale bar: 20 µm.

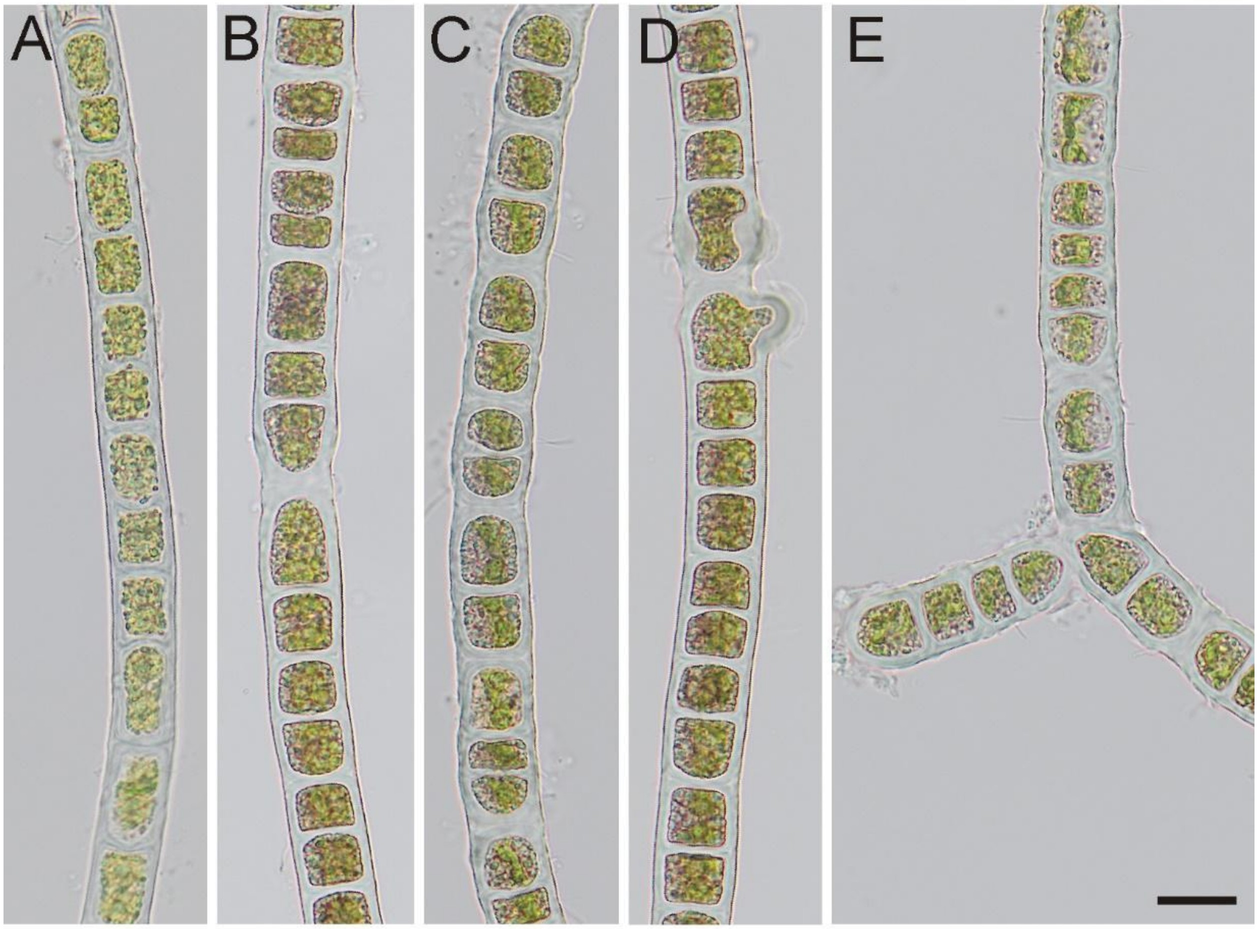

Figure S7 *Zygogonium* cf. *ericetorum* Group 1B filaments from Tyrol, Ellmau **(A-E)**; (A, E) vegetative filaments, (B-D) filaments with oval akinetes with one or two chloroplasts, (E) filament with side branch; Scale bar: 20 µm.

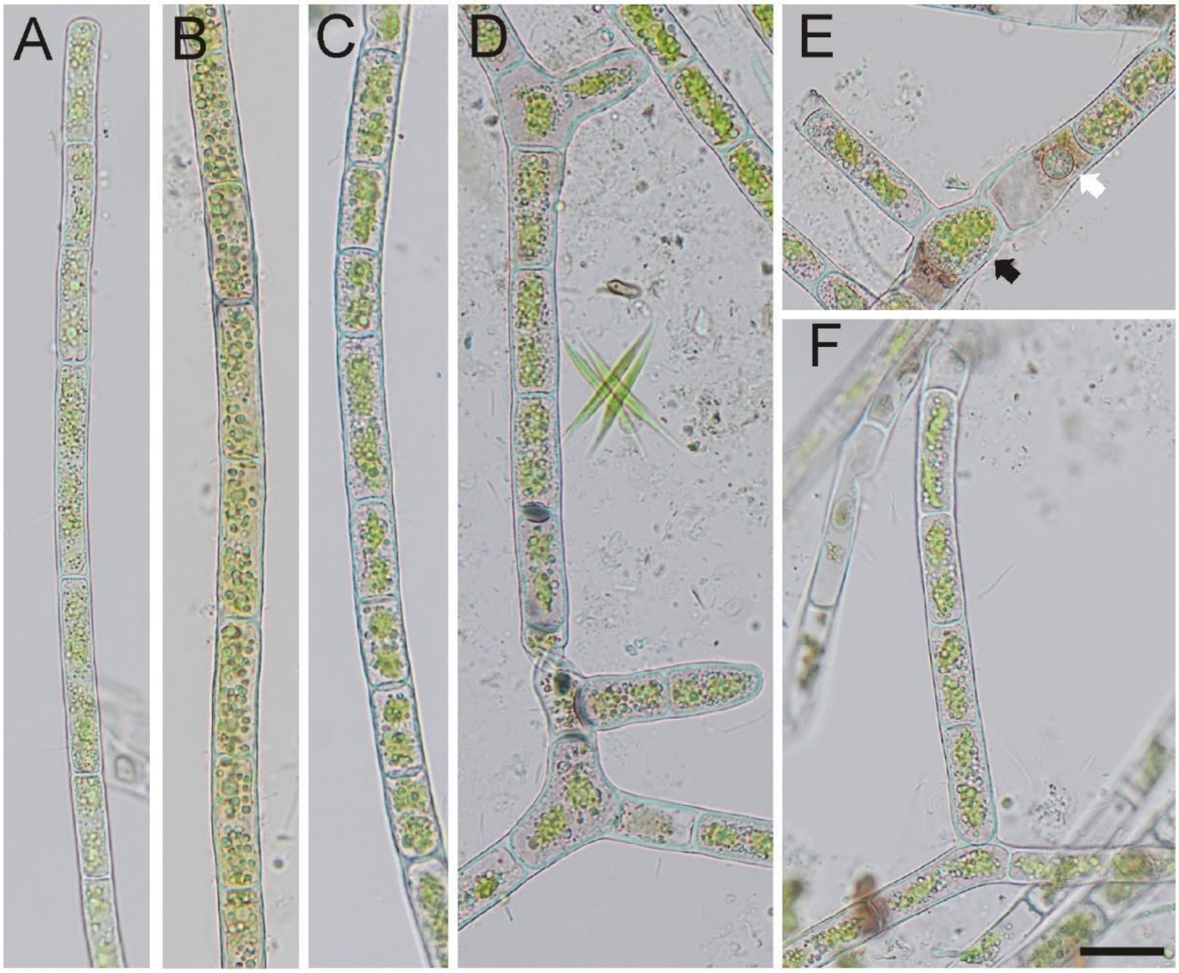

Figure S8. *Zygogonium angustum,* sp. nov. Group 2 filaments from Ireland 2-6 **(A, C, D, F)** and Ireland 4-7 **(B, E** - not sequenced); (A-C, D, F) vegetative filaments, (E) filament with an aplanospore inside the branching cell (black arrow) and parasite cyst inside another cell (white arrow), (D-F) filament with side branches; scale bar: 20 µm.

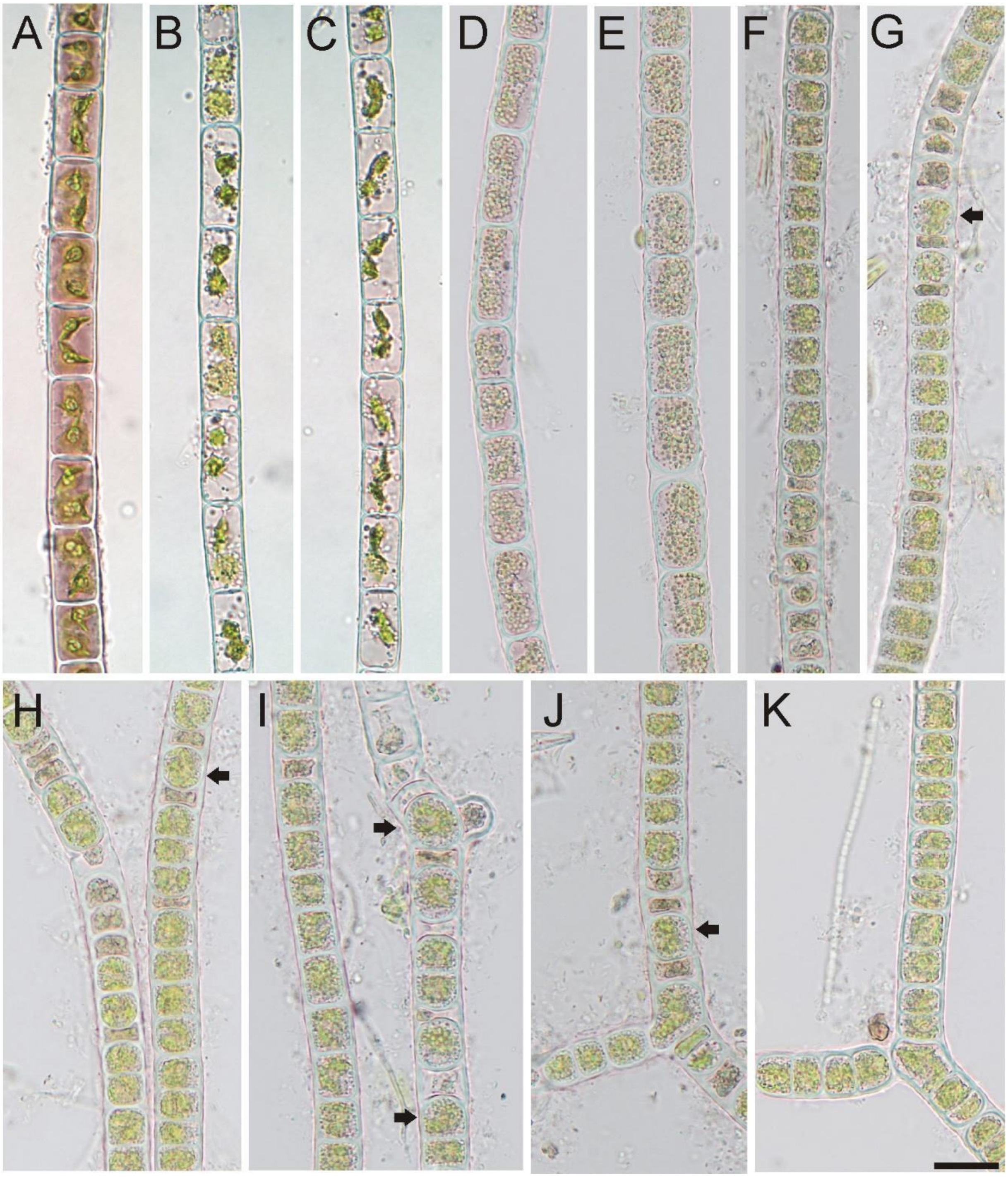

Figure S9 *Zygogonium* cf. *ericetorum* Group 3 filaments from Tantalus Creek in Yellowstone National Park, USA **(A-C)** and Norway 1 **(D-K)**; (A – F, K) vegetative filaments, (G-J) filaments with aplanospores (arrows), (I-K) filament with side branches, note the formation of aplanospore inside the branching cell (I arrow); Scale bar: 20 µm.

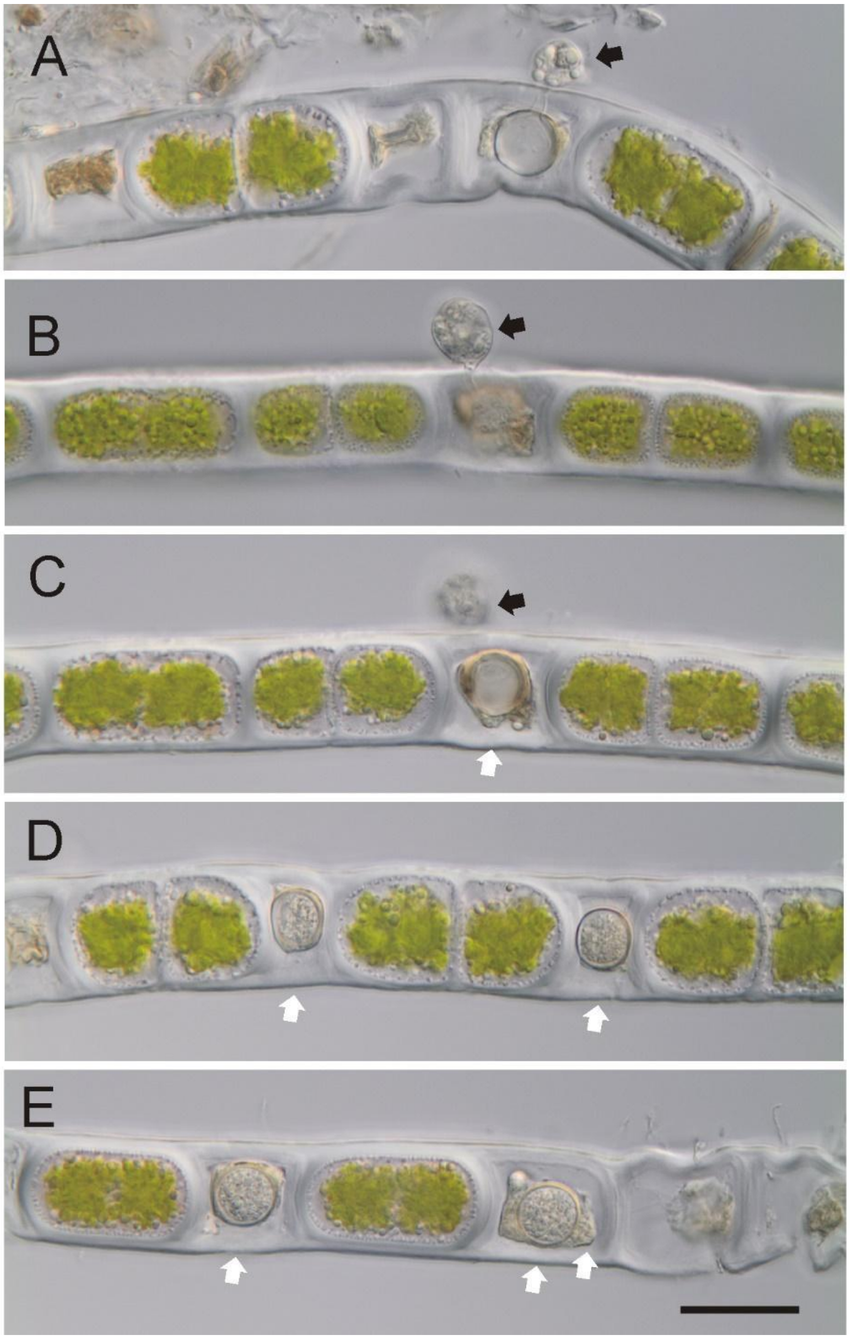

Figure S10 Fungal parasite on *Zygogonium* cf. *ericetorum* Group 1B from Ellmau, Tyrol; (A-C) extracellular sporangium of chytrid parasite (black arrows) on the top of *Zygogonium* filaments, (C-E) Intracellular parasite cysts (white arrows) inside *Zygogonium* cells missing chloroplasts; Images obtained by differential interference contrast light microscopy; Scale bar: 20 µm.
