# Supplementary material for: Genetic and morphological variation in the genus Zygogonium (Zygnematophyceae, Charophyta) from localities in Europe and North America and description of Z. angustum, sp. nov
Source: J Phycol. 2025 Apr 9;61(3):587–606. doi: 10.1111/jpy.70012 (PMC12168101; doi:10.1111/jpy.70012)
Supplement: Supplementary file 2 — Table S1. Sequence data availability of the Zygogonium collections used in this study and of outgroup taxa for phylogenetic analyses. DNA numbers correspond to the DNA number in Tables 1–2, with JH corresponding to collections by John Hall. NCBI accession numbers for the new sequences are shown in boldface font, and these are followed by the length of the determined sequence in nt, shown in parenthesis. ND = sequence not determined during our study; n/a = published sequence not available. [file JPY-61-587-s001.pdf]

**Supplementary Table:**

# Genetic and morphological variation in the genus *Zygogonium* (Zygnematophyceae, Charophyta) from localities in Europe and North America and description of a new species *Z. angustum*, sp. nov.

*Journal of Phycology*

Rosalina Stancheva[1], Louise A. Lewis[2], John Hall[3], Tereza Šoljaková[4], Charlotte Permann[5], Andreas Holzinger[5*]

*Author for correspondence: Andreas.Holzinger@uibk.ac.at

**Table S1** Sequence data availability of the *Zygogonium* collections used in this study and of outgroup taxa for phylogenetic analyses. DNA numbers correspond to the DNA number in Tables 1-2, with JH corresponding to collections by John Hall. NCBI accession numbers for the new sequences are shown in boldface font, and these are followed by the length of the determined sequence in nt, shown in parenthesis. ND=sequence not determined during our study; n/a=published sequence not available.

| DNA / culture collection # | Species | *psb*C accession (length, nt) | *atp*B accession (length, nt) | *rbc*L accession (length, nt) |
|---|---|---|---|---|
| JH1396 | *Zygogonium ericetorum* | KM205112 | KM205114 | KM205115 |
| JH1397 | *Zygogonium ericetorum* | KM205113 | PP993494 (468) | KM205116 |
| 1477 | *Zygogonium ericetorum* | PQ044409 (1222) | PP993495 (587) | ND |
| 1479 | *Zygogonium* cf. *ericetorum* | PQ044410 (1222) | PP993496 (535) | PP993480 (729) |
| 1480 | *Zygogonium* cf. *ericetorum* | PQ044411 (1222) | PP993497 (528) | PP993481(708) |
| 1482 | *Zygogonium* cf. *ericetorum* | PQ044412 (1216) | ND | ND |
| 1484 | *Zygogonium angustum* | PQ044413 (746) | PP993498 (537) | PP993482 (727) |
| 1490 | *Zygogonium angustum* | PQ044430 (1214) | PP993499 (486) | PP993483 (765) |
| 1491 | *Zygogonium* cf. *ericetorum* | PQ044414 (1214) | PP993500 (1228) | PP993484 (704) |

| | | | | |
|---|---|---|---|---|
| 1496 | *Zygogonium angustum* | PQ044415 (1214) | ND | ND |
| 1498 | *Zygogonium angustum* | PQ044416 (1220) | PP993501 (596) | PP993485 (1269) |
| 1499 | *Zygogonium ericetorum* | PQ044417 (1214) | PP993502 (1043) | PP993486 (1277) |
| 1506 | *Zygogonium ericetorum* | PQ044418 (1215) | ND | PP993487 (1230) |
| 1507 | *Zygogonium* cf. *ericetorum* | PQ044419 (1211) | ND | PP993488 (624) |
| 1508 | *Zygogonium* cf. *ericetorum* | PQ044420 (1215) | ND | PP993489 (1214) |
| 1509 | *Zygogonium ericetorum* | PQ044421 (1211) | ND | PP993490 (700) |
| 1510 | *Zygogonium ericetorum* | PQ044431 (1214) | ND | PP993491 (355) |
| 1516 | *Zygogonium ericetorum* | PQ044422 (1227) | ND | PP993492 (783) |
| 1517 | *Zygogonium ericetorum* | PQ044423 (1212) | ND | ND |
| 1518 | *Zygogonium ericetorum* | PQ044424 (1210) | PP993503 (589) | ND |
| 1519 | *Zygogonium ericetorum* | PQ044425 (1212) | ND | PP993493 (991) |
| 1520 | *Zygogonium ericetorum* | PQ044432 (1214) | PP993504 (1186) | ND |
| 1521 | *Zygogonium ericetorum* | PQ044433 (1215) | PP993505 (1186) | ND |
| 1470 | *Zygogonium* cf. *ericetorum* | PQ044426 (1151) | ND | ND |
| 1471 | *Zygogonium* cf. *ericetorum* | PQ044427 (1113) | ND | ND |
| 1472 | *Zygogonium* cf. *ericetorum* | PQ044434 (1132) | ND | ND |
| 1473 | *Zygogonium* cf. *ericetorum* | PQ044428 (707) | ND | PP993479 (504) |
| 1475 | *Zygogonium* cf. *ericetorum* | PQ044429 (1078) | ND | ND |
| outgroup taxa | | | | |
| SAG 698-1a | *Zygnema circumcarinatum* | NC_008117 | NC_008117 | NC_008117 |
| SAG 698-2 | *Zygnema cylindricum* | EF371357 | KC779158 | KM205108 |

| | | | | |
|---|---|---|---|---|
| UTCC 136 | *Zygnema tunetanum* | JQ780057 | KC779162 | KM205110 |
| JH0031 | *Mesotaenium* sp. | EF371310.2 | KC779081 | EF371310 |
| GSM.2.16.l | Zygnematophyceae sp. | OM241459.1 | N/A | N/A |
| KM.19b | Zygnematophyceae sp. | OM241458.1 | N/A | N/A |
